# One-Hot Multi-Level Leaky Integrate-and-Fire Spiking Neural Networks for Enhanced Accuracy-Latency Tradeoff

**DOI:** 10.1109/access.2025.3546508

**Published:** 2025-02-27

**Authors:** PIERRE ABILLAMA, CHANGWOO LEE, ANDREA BEJARANO-CARBO, QIRUI ZHANG, DENNIS SYLVESTER, DAVID BLAAUW, HUN-SEOK KIM

**Affiliations:** Department of Electrical Engineering and Computer Science, University of Michigan, Ann Arbor, MI 48109, USA

**Keywords:** Spiking neural networks, one-hot, multi-level, low latency, energy efficiency

## Abstract

Spiking neural networks (SNNs) hold significant promise as energy-efficient alternatives to conventional artificial neural networks (ANNs). However, SNNs require computations across multiple timesteps, resulting in increased latency, heightened energy consumption, and additional memory access overhead. Techniques to reduce SNN latency down to a unit timestep have emerged to realize true superior energy efficiency over ANNs. Nonetheless, this latency reduction often comes at the expense of noticeable accuracy degradation. Therefore, achieving an optimal balance in the tradeoff between accuracy and energy consumption by adjusting the latency of multiple timesteps remains a significant challenge. This work leverages an additional dimension to enhance the accuracy-energy tradeoff space using a novel *one-hot multi-level leaky integrate-and-fire* (M-LIF) neuron model. The proposed one-hot M-LIF model represents the inputs and outputs of hidden layers as a set of one-hot binary-weighted spike lanes to find better tradeoff points while still being able to model conventional SNNs. For image classification on static datasets, we demonstrate one-hot M-LIF SNNs outperform iso-architecture conventional LIF SNNs in terms of accuracy (2% higher than VGG16 SNN on ImageNet) while still being energy-efficient (20× lower energy than VGG16 ANN on ImageNet). For dynamic vision datasets, we demonstrate the ability of M-LIF SNNs to reduce latency by 3× compared to conventional LIF SNNs while limiting accuracy degradation (< 1%).

## I. INTRODUCTION

Neural networks have become a fundamental technique for solving many important problems such as image classification, object detection, and face recognition [1], [2]. As neural network accuracy improves, models become increasingly complex, making their energy-efficient deployment on the edge a significant challenge. In order to reduce the computational complexity of these tasks, spiking neural networks (SNNs) [3] were proposed as an alternative to traditional artificial neural networks (ANNs) [1], [4]. SNNs infer inputs across multiple timesteps while ANNs perform a one-shot inference, essentially over a single timestep. Neurons in SNNs differ from those in ANNs as they operate on sparse binary spike trains as opposed to non-binary ‘analog’ activations, resulting in the substitution of multiplications with energy-efficient additions [5].

To model spikes over time, SNNs employ various techniques, most notably the leaky integrate-and-fire (LIF) neuron model [6], [7]. Each neuron is characterized by two parameters: firing threshold and membrane leakage. During a timestep, a neuron either remains silent or produces a spike if the membrane potential exceeds its firing threshold. The membrane potential can shrink over time depending on the membrane leakage and is reset if a spike is produced. Using such models, many training methods have emerged and can be categorized into two main approaches: ANN-SNN conversion and direct training. ANN-SNN conversion methods [8], [9], [10] convert the weights of a pre-trained ANN to an iso-architecture SNN. However, these methods can require a large number of timesteps (on the order of 1000 in some cases [11]) to achieve comparable or better accuracy than ANNs. Note that multi-timestep inference results in more memory storage/accesses which can dominate the compute cost [12]. Therefore, the reliance on multi-timestep processing in ANN-to-SNN conversion has been a key factor hindering the practical deployment of SNNs in energy-constrained edge scenarios.

Direct training using surrogate gradient-based optimization and back-propagation through time (BPTT) [13], [14], [15] has enabled training SNNs with significantly fewer timesteps, occasionally reducing them to just a single timestep [16]. However, these SNNs still lag ANNs in terms of accuracy. For image classification on static datasets [1], [17], SNNs cannot bridge this accuracy gap with ANNs without increasing the number of timesteps, which in turn reduces energy efficiency. Moreover, single-timestep SNNs such as [16] require iterative temporal pruning to converge, rendering training more time-consuming. For image classification, multi-timestep SNNs traditionally outperform ANNs on dynamic vision sensor [18] data but suffer from a sharp accuracy degradation [19] with a reduced number of timesteps.

**FIGURE 1.**
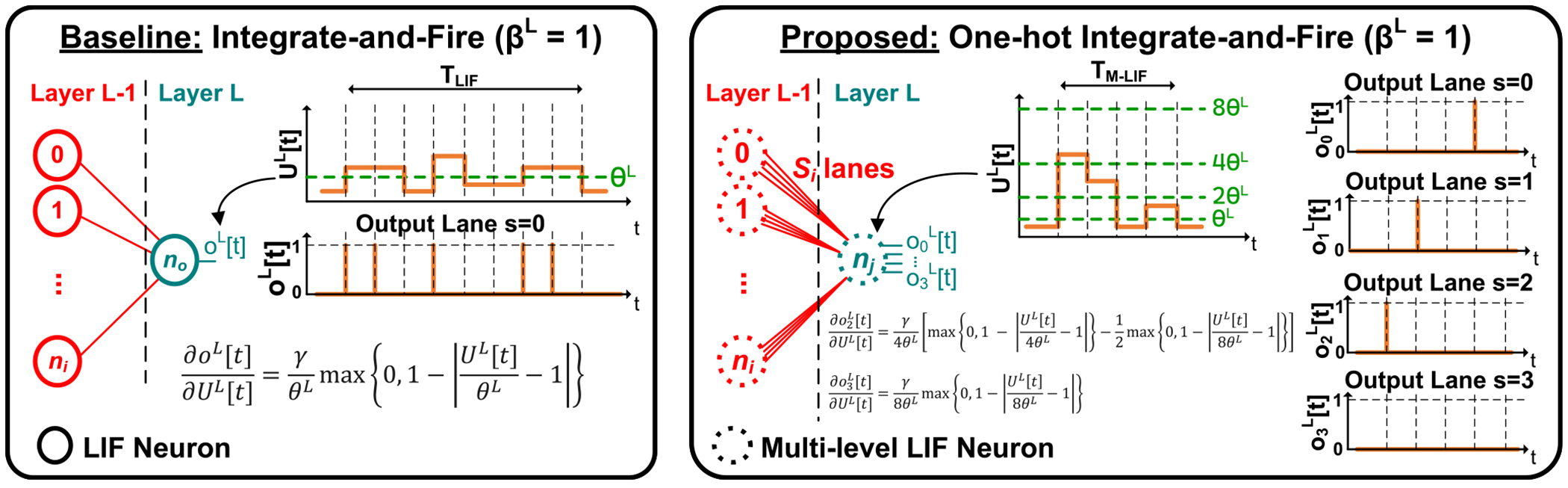
LIF neuron model (left) vs. one-hot M-LIF neuron model with *S_o_* = 4 lanes (right).

As conventional LIF models employ binary-valued neuron spike outputs, traditional SNNs are restricted to solely scaling the temporal dimension *T* (i.e., the number of timesteps) to achieve different accuracy-energy efficiency tradeoffs. Prior SNN works have explored the interaction between activation bit-width and timesteps using multi-spike [20], [21] and multi-threshold [22], [23] neurons to increase activation precision via uniform quantization. However, these approaches raise computational energy costs by disrupting the multiplication-free nature of traditional SNNs. To address these limitations and enhance the accuracy-energy tradeoff space, we extend the range of neuron spike outputs to include strictly powers of two by leveraging an additional dimension, *S*, and by using a novel *one-hot multi-level leaky integrate-and-fire* (M-LIF) neuron model as illustrated in Figure 1. The proposed one-hot M-LIF model has the following key properties: 1) it uses the *S* dimension to represent hidden layer outputs (inputs) as a set of *S_o_* (*S_i_*) binary-weighted spike lanes, and 2) it limits the simultaneous firing behavior of those *S_o_* (*S_i_*) spike lanes to only a single lane per timestep. These two properties of our one-hot M-LIF model enable new accuracy-energy tradeoff points for SNNs while still only requiring additions (without multiplications) like the conventional LIF model. Furthermore, the proposed one-hot M-LIF model can be easily integrated into prior existing training frameworks. For static datasets such as CIFAR and ImageNet, we demonstrate that one-hot M-LIF SNNs outperform conventional LIF SNN accuracy while achieving better or comparable energy efficiency on various architectures including the high-performance spike-driven transformer [15]. For dynamic vision datasets such as DVS-CIFAR10 [24], [25], we show that M-LIF SNNs using multi-level input layer encoding can achieve reduced timesteps (energy consumption) compared to conventional LIF SNNs for comparable or better accuracy. To summarize, the main contributions of this paper are:

- We propose a new direction to balance SNN energy efficiency and accuracy using a novel one-hot multi-level leaky-integrate-and-fire (M-LIF) neuron model.
- We enable a new tradeoff and show that one-hot M-LIF SNNs are more accurate than iso-architecture LIF SNNs while consuming comparable or lower energy with fewer timesteps.
- We demonstrate the benefit of one-hot M-LIF SNNs with dynamic vision sensor-based input compared to conventional SNNs. To the best of our knowledge, this is the first SNN work to achieve a top-1 accuracy of 82.5% on DVS-CIFAR10 using only 3 timesteps.

## II. BACKGROUND AND RELATED WORKS

### A. LEAKY INTEGRATE-AND-FIRE MODEL

A conventional spiking neural network (SNN) layer under the leaky integrate-and-fire (LIF) neuron model is described by

(1) $$u^{l}[t]=\beta^{l}u^{l}[t-1]+W^{l}o^{l-1}[t]-\theta^{l}o^{l}[t-1]$$

(2) $$o^{l}[t]=\{\begin{array}{ll} 1, & \text{if}\;1<\frac{u^{l}[t]}{\theta^{l}}\\ 0, & \text{otherwise} \end{array}$$

where $W^{l}$ is the weight matrix connecting layers l-1 and l,u is a vector containing the membrane potential of output neurons, β∈[0,1] is the leakage factor, o is an output vector of binary spikes, θ>0 is the firing threshold, and t∈{0,1,2,…} represents the discrete timestep. The first term in Equation (1) corresponds to the membrane leakage allowing the potential to shrink (leak) over time, and the final term accounts for resetting the potential to a specific value when an output binary spike given by Equation (2) is generated. All neurons in an input/hidden layer typically share the same leakage factor and firing threshold values. As for the final layer, adopting the LIF model without any modifications can significantly impact the accuracy [13], [14]. Hence, the final output layer neurons only accumulate incoming inputs without any leakage and do not fire output spikes. Finally, the inference process is repeated for T timesteps from t=0 to T-1, and the output of the last layer is averaged to produce the final result. The output spiking behavior of traditional LIF neurons in Equation (2) is depicted in Figure 1 (left) where the output range of the membrane potential $u^{L}[t]$ is subdivided using a single decision boundary defined by the threshold $\theta^{L}$.

### B. ANN-SNN CONVERSION

ANN-SNN conversion methods [8], [9], [10], [11], [26], [27] convert the weights of a pre-trained ANN to an iso-architecture SNN. Specifically, these methods convert the output of a rectified linear unit neuron in an ANN into a sequence of binary spikes in the SNN over multiple timesteps. The primary challenge in this technique is determining the firing threshold in such a way that it balances the accuracy-latency tradeoff. In these methods, the firing thresholds are generally determined by profiling the pre-trained ANN and recording a certain percentile of layers’ input activation distributions. However, these heuristic techniques can lead to a sub-optimal choice of firing threshold and can also require a large number of timesteps to achieve comparable or better accuracy than ANNs, thus further aggravating the accuracy-latency tradeoff. This multi-timestep processing requirement is a challenge for widespread SNN deployment as it primarily introduces more memory storage and accesses which can be significantly higher than compute cost [12].

### C. DIRECT TRAINING

An alternate approach to training SNNs is to use gradient-based techniques, such as back-propagation, either from scratch or from a pre-trained iso-architecture ANN [13], [14], [16], [28]. These approaches relate the temporal dimension of SNNs to that of recurrent neural networks, and perform back-propagation through time (BPTT) to learn weights across multiple timesteps.

**FIGURE 2.**
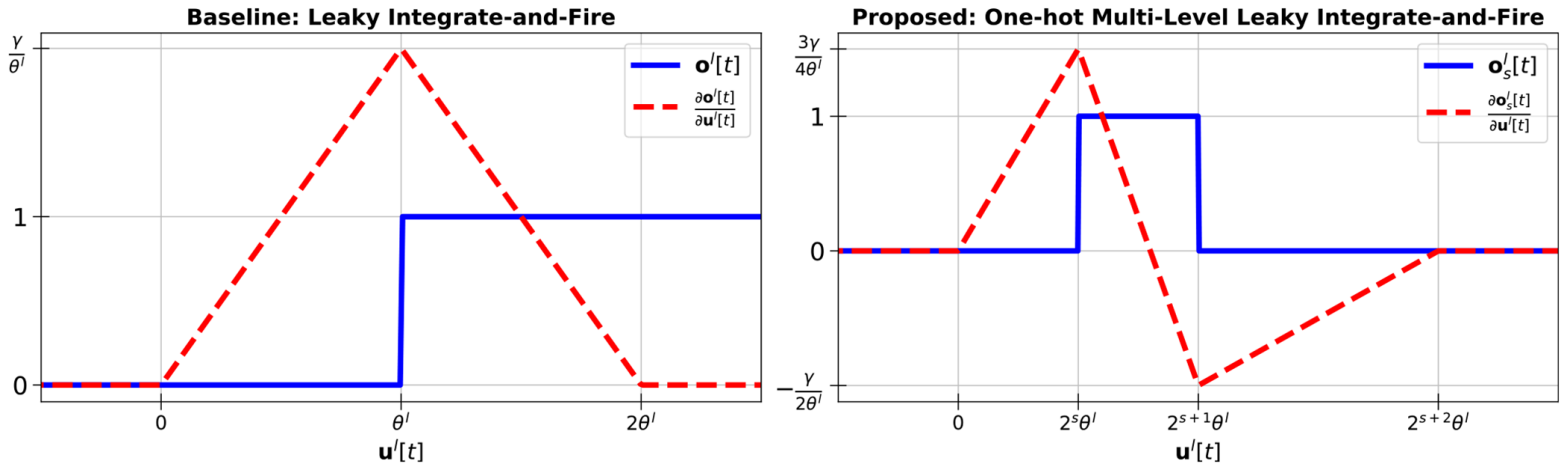
Output activation and surrogate gradient functions for conventional LIF neuron model (left) and one-hot multi-level LIF neuron model for 0 ≤ *s* < *S_o_* − 1 (right).

The cross-entropy loss L and gradients $\partial L/\partial W^{l}$ are calculated by

(3) $$L=-\underset{i}{\sum }y_{i}\text{log}\left(\text{Φ}{\left(o^{L}[T-1]\right)}_{i}\right)$$

(4) $$\frac{\partial L}{\partial W^{l}}=\underset{t}{\sum }\frac{\partial L}{\partial o^{l}[t]}\frac{\partial o^{l}[t]}{\partial u^{l}[t]}\frac{\partial u^{l}[t]}{\partial W^{l}}$$

where L is the index of the final layer, Φ(·) denotes the softmax function, and y is the one-hot encoded vector of the true label. The term $\partial o^{l}[t]/\partial u^{l}[t]$ in Equations (3) and (4) is the discontinuous gradient that is typically replaced by differentiable surrogate gradients. Prior works have explored the use of various surrogate gradient shapes such as triangular [13], [29], or the derivative of the sigmoid function [15] which are given below in Equations (5) and (6), respectively, where γ and α are constants used to scale the shapes of the gradients. The triangular surrogate gradient in Equation (5) used for the output spike of the traditional LIF model is depicted in Figure 2 (left) for the single activation channel case where γ is shown to scale the peak value of the gradient at $u^{L}[t]=\theta^{L}$.

(5) $$\frac{\partial o^{l}[t]}{\partial u^{l}[t]}=\text{diag}\left(\frac{\gamma }{\theta^{l}}\text{max}\left\{0,1-\left|\frac{u^{l}[t]}{\theta^{l}}-1\right|\right\}\right)$$

(6) $$\frac{\partial o^{l}[t]}{\partial u^{l}[t]}=\text{diag}\left(\frac{\alpha }{\theta^{l}}\left(1-\sigma \left(\alpha \left(\frac{u^{l}[t]}{\theta^{l}}-1\right)\right)\right)\cdot \sigma \left(\alpha \left(\frac{u^{l}[t]}{\theta^{l}}-1\right)\right)\right)$$

Compared to ANN-SNN conversion techniques, direct training approaches typically achieve a better accuracy-latency tradeoff (higher accuracy using fewer timesteps overall) at the cost of more compute- and memory-intensive training [30]. To achieve competitive accuracy, most employ direct input encoding [31] and utilize the first layer as a spike generator by directly feeding pixel values as inputs to the network. Through gradient-based learning, many recent works have sought to optimize different aspects of this training methodology such as loss function definition [14], initialization and parameterization [13], [16], and extension to advanced network architectures such as spike-driven transformers [15]. Notably, the authors in [16] propose temporal pruning to gradually reduce the number of timesteps to successfully train SNNs with as little as a single timestep, despite a noticeable accuracy degradation (up to 4%). All these works differ from ours as they are restricted to solely scaling the temporal dimension *T* in order to achieve different accuracy-latency tradeoffs given a fixed neural network architecture. To address this limitation, our approach leverages the *S* dimension using our one-hot multi-level LIF (M-LIF) neuron model to improve the accuracy-latency tradeoff space.

### D. QUANTIZED-ACTIVATION ANNS

As the number of timesteps converges to one, conventional SNNs become closely related to binary activated artificial neural networks (BNNs) [32], [33] as both use binary activations to perform an inference over a single timestep. The authors in [16] discuss that they are in fact distinct. While SNNs quantize outputs to spikes (*i.e.* {0, 1}), BNNs quantize outputs to be ±1. Unlike BNNs which use non-linear activation functions where the firing threshold is zero, the firing threshold is learnable in SNNs. The authors in [16] observe that this allows SNNs to outperform BNNs in terms of accuracy and scale better to larger datasets such as ImageNet. Moreover, LIF enables SNNs to extend the same network for sequential processing unlike BNNs. Similarly, our one-hot M-LIF SNNs become closely related to log-quantized-activation ANNs (LQ-ANNs) [34], [35] as timesteps converge to one, but remain distinct for analogous reasons. A discussion regarding similarities and differences is provided in Section III-B.

### E. QUANTIZED-ACTIVATION SNNS

Prior SNN works have explored the interaction between activation bit-width and timesteps [20], [21], [22], [23], [36], [37]. Our work’s major distinction is that the one-hot M-LIF neuron model constrains outputs of neurons to be powers-of-two while enabling better accuracy with low spike rates in the low-latency (unit-timestep) regime.

Multi-threshold SNNs [22], [23] apply multiple thresholds to each membrane potential after integration, either in parallel or sequentially, and sum resulting spikes together to produce an output activation. As a result, multi-threshold neurons lead to a uniformly quantized output whose output range depends on the number of thresholds used. Our one-hot M-LIF neuron on the other hand uses dual-sided thresholding as shown later in Equation (8) to only produce a single spike on one of *S_o_* weighted output spike lanes. As a result, we restrict outputs to be strictly powers-of-two, a constraint which is accounted for during training. This enables efficient FP32 weight exponent updates via a single INT8 addition as discussed in Section IV, unlike the multiplication demands of uniformly quantized outputs. In [22], [23], the surrogate gradient of a traditional LIF neuron (e.g., Equations (5) or (6)) is applied to each spike lane while in our approach, the surrogate gradient of the traditional LIF neuron was adapted to handle dual-sided thresholding per spike lane. Finally, unlike [22], [23] which perform evaluations solely on convolutional neural network (CNN) architectures, our evaluations span both CNNs and the more complex, high-performing spike-driven transformer architecture.

Burst-spike neuron models [36], [37] work by increasing the spike rate, in other words the number of spikes issued within a single timestep. This is unlike our approach which emits at most a single spike per timestep independent of the number of spike lanes used per neuron. Moreover, evaluations for burst-spike neuron models show that resulting SNNs do not scale down to unit-timestep processing. For example, [37] reports results for at least *T* ≥ 32 timesteps with a 14.2% and 18.2% accuracy drop at *T* = 16 for VGG16 and ResNet-20 on CIFAR100, respectively. The work in [36] reports an accuracy of 70.61% for VGG16 on ImageNet with *T* = 8 (vs. 71.05% with *T* = 1 for our one-hot M-LIF VGG16 SNN using *T* = 1 and *S* = 3) and lacks comprehensive reporting of energy estimation analyses for all experimental results. Memory energy models for SNNs such as in [38] indicate that memory access energy of spikes and membrane potentials scales linearly with the number of timesteps, with single memory access costs often being 10× higher than single compute energy costs as discussed in [12] for a 45nm CMOS technology. Unlike burst-spike neuron models that require multi-timestep processing, our model achieves high accuracy with unit-timestep processing as reported later in Table 1 of our manuscript along with comparisons against existing state-of-the-art unit-timestep SNNs [16], [38].

## III. PROPOSED ONE-HOT MULTI-LEVEL LIF-BASED SNNS

### A. MULTI-LEVEL LIF MODEL

Our goal is to reduce the number of timesteps T during SNN inference while improving accuracy and maintaining the low-spike rates of traditional SNNs. By reducing the timesteps T while maintaining low spike rates, we can consequently decrease the energy overhead associated with multi-timestep processing. To do so, our multi-level leaky integrate-and-fire (M-LIF) neuron model still uses a single membrane potential per output neuron while extending the range of neuron outputs to more than just binary representations. It employs a new dimension S to represent hidden layer outputs (inputs) as a set of $S_{o}\left(S_{i}\right)$ binary-weighted spike lanes. We denote $o_{s}^{l}[t]$ to be the output vector of binary spike lane s in layer l at timestep t. Each spike lane s is weighted by $2^{s}$ resulting in a non-binary output range for the neuron output $o^{l}[t]$. As a result, Equation (1) needs to be modified to combine weighted spike lanes prior to updating the membrane potential as follows

(7) $$u^{l}[t]=\beta^{l}u^{l}[t-1]+\overset{S_{i}-1}{\underset{s=0}{\sum }}2^{s}W^{l}o_{s}^{l-1}[t]-\overset{S_{o}-1}{\underset{s=0}{\sum }}2^{s}\theta^{l}o_{s}^{l}[t-1]\;=\beta^{l}u^{l}[t-1]+W^{l}o^{l-1}[t]-\theta^{l}o^{l}[t-1].$$

We also define $\omega \leq S_{o}$ as the maximum number of simultaneously firing output spike lanes in any given timestep. With all spike lanes sharing the same firing threshold and membrane potential, it is non-trivial to devise a firing mechanism with ≤ω concurrent firing lanes. Section III-B discusses the practical one-hot case we propose for SNNs where ω=1.

### B. ONE-HOT MULTI-LEVEL LIF MODEL

In the one-hot M-LIF model, ω=1 and *only one of the* $S_{o}$ *output spike lanes fires in any given timestep*. The threshold mechanism is given by

(8) $$o_{s}^{l}[t]=\{\begin{array}{ll} 1, & \text{if}\;\left(2^{s}<\frac{u^{l}[t]}{\theta^{l}}\leq 2^{s+1}\wedge 0\leq s<S_{o}-1\right)\vee \left(2^{s}<\frac{u^{l}[t]}{\theta^{l}}\wedge s=S_{o}-1\right)\\ 0, & \text{otherwise} \end{array}$$

Figure 1 depicts the difference between the conventional LIF model (left) and our proposed one-hot (ω=1) M-LIF model (right) given $S_{o}=4$. Instead of having only one output (input) spiking signal, an M-LIF neuron has multiple ($S_{o}=4$) binary-weighted output (input) spike lanes. With the one-hot constraint, only a single spike lane fires at any given timestep. In the example illustrated in Figure 1 (right), the membrane potential can increase by one of $S_{o}=4$ possible levels in $\left\{\theta^{l}\right.,\left.2\theta^{l},4\theta^{l},8\theta^{l}\right\}$ from one timestep to the next, as opposed to the single level $\theta^{l}$ in the conventional LIF model. The output spike lanes are one-hot, meaning that the output range is also subdivided into $S_{o}=4$ non-overlapping decision boundaries using the binary weight of each spiking lane as shown in Equation (9). Therefore, for a single activation channel case, we have $\sum_{s=0}^{S_{o}-1}\;2^{s}o_{s}^{l}[t]=o^{l}[t]\in \left\{0,1,2,4,\ldots ,2^{S_{o}-1}\right\}$.

(9) $$o_{0}^{l}[t]=\{\begin{array}{ll} 1, & \text{if}\;1<\frac{u^{l}[t]}{\theta^{l}}\leq 2\\ 0, & \text{otherwise} \end{array}\;o_{1}^{l}[t]=\{\begin{array}{ll} 1, & \text{if}\;2<\frac{u^{l}[t]}{\theta^{l}}\leq 4\\ 0, & \text{otherwise} \end{array}\;o_{2}^{l}[t]=\{\begin{array}{ll} 1, & \text{if}\;4<\frac{u^{l}[t]}{\theta^{l}}\leq 8\\ 0, & \text{otherwise} \end{array}\;o_{3}^{l}[t]=\{\begin{array}{ll} 1, & \text{if}\;8<\frac{u^{l}[t]}{\theta^{l}}\\ 0, & \text{otherwise} \end{array}$$

Note that by setting $S_{i}=S_{o}=1$ (i.e., single lane), Equations (7) and (8) simplify to Equations (1) and (2). Therefore, binary spiking SNNs can be considered as a special case of the proposed one-hot M-LIF scheme.

#### 1) SURROGATE GRADIENT FOR BACK-PROPAGATION TRAINING

**FIGURE 3.**
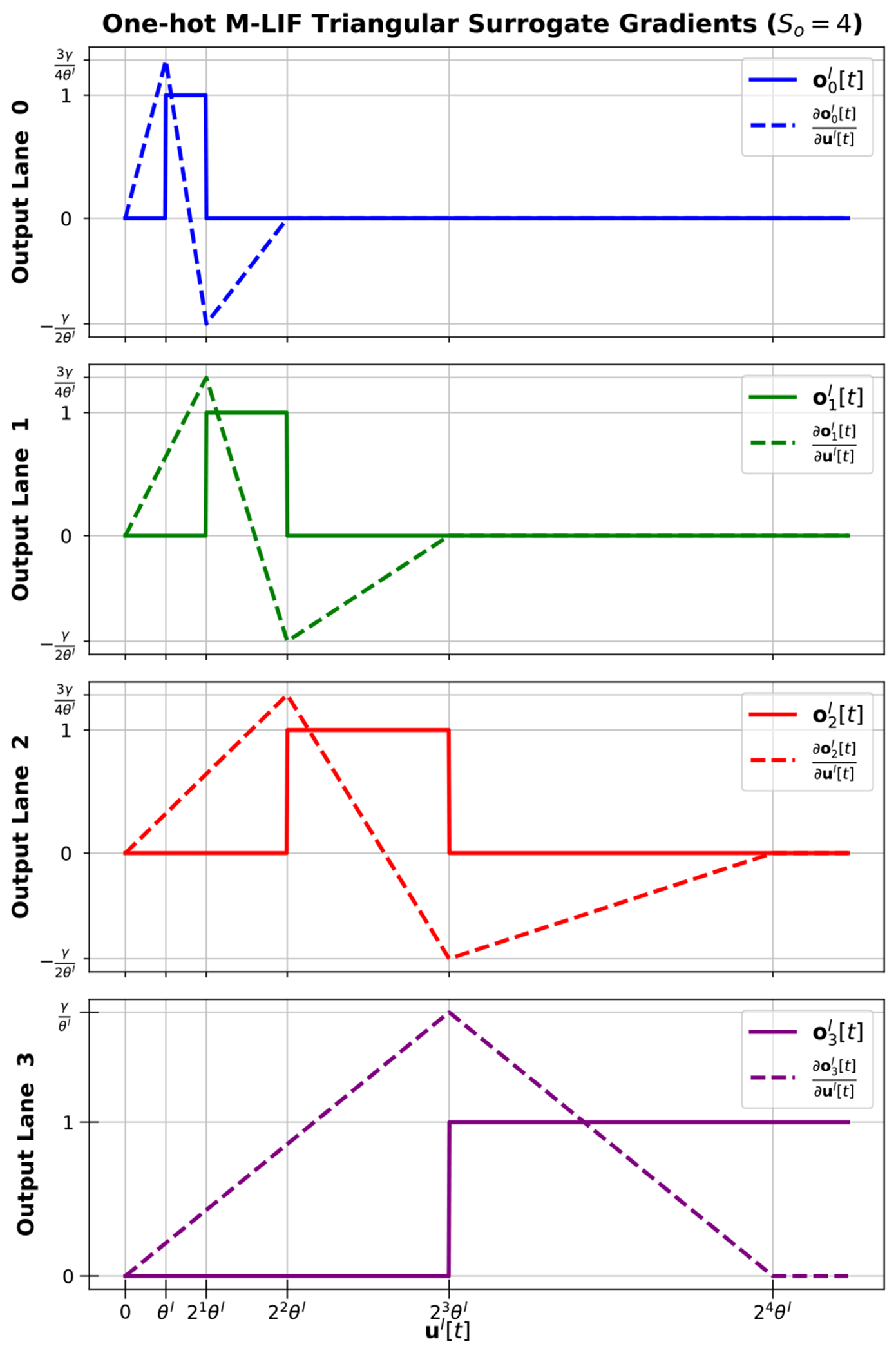
Example of triangular surrogate gradient for *S_o_* = 4 one-hot M-LIF neuron output spike lanes.

Given the update to $o^{l}[t]$ in Equation (8), the surrogate gradient is extended from the LIF case to the proposed one-hot M-LIF neurons. For example, Figure 2 illustrates the differences in an updated triangular surrogate gradient for the single activation channel case when $0\leq s<S_{o}-1$. Each spike lane is now a window function of $u^{l}[t]$ as opposed to a step function. This is equivalent to the difference of two step functions, the first of which is evaluated at the spike lane’s threshold, and the second of which is evaluate at the next spike lane’s threshold. As a result, the surrogate gradient becomes the difference of two triangular sub-gradients, one for each of the rising and falling window edges, as shown in Equation (10). An example illustrating Equation (10) for $S_{o}=4$ is provided in Figure 3. An additional example for the derivative of the sigmoid function is included in Appendix A.


$$z^{l}(s)=\frac{\gamma }{2^{s}\theta^{l}}\text{max}\left\{0,1-\left|\frac{u^{l}[t]}{2^{s}\theta^{l}}-1\right|\right\}$$

$$\frac{\partial o^{l}[t]}{\partial u^{l}[t]}=\overset{S_{o}-1}{\underset{s=0}{\sum }}2^{s}\frac{\partial o_{s}^{l}[t]}{\partial u^{l}[t]}$$

(10) $$\frac{\partial o_{s}^{l}[t]}{\partial u^{l}[t]}=\{\begin{array}{ll} \text{diag}\left(z^{l}(s)\right), & \text{if}\;s=S_{o}-1\\ \text{diag}\left(\overset{1}{\underset{a=0}{\sum }}{\left(-1\right)}^{a}z^{l}(s+a)\right), & \text{if}\;0\leq s<S_{o}-1 \end{array}$$

#### 2) DISCUSSION

As T→1 (i.e., unit timestep inference), Equations (7) and (8) can be rewritten as

(11) $$u^{l}=\overset{S_{i}-1}{\underset{s=0}{\sum }}2^{s}W^{l}o_{s}^{l-1}=W^{l}o^{l-1}$$

where

$$o^{l}=2^{\text{clip}\left(\left\lfloor {\text{log}}_{2}\left(\frac{u^{l}}{\theta^{l}}\right)\right\rfloor ,0,S_{o}\right)},$$

$$\text{clip}(x,v,z)=\{\begin{array}{ll} -\infty , & \text{if}\;x\leq v\\ z-1, & \text{if}\;x\geq z\\ x, & \text{otherwise.} \end{array}$$

From this, an observable parallel can be drawn between our one-hot M-LIF SNNs with unit timestep (T=1) and log quantized-activation ANNs (LQ-ANNs) [39], [40]. While one-hot M-LIF-based SNNs are trained in a single phase, LQ-ANN training is performed in two phases per epoch. First, using the entire training dataset and full precision inference, a percentile value α of each layer’s input activation distribution is recorded. Second, a straight-through estimator is typically applied to approximate the gradient with respect to quantized activations. Using b bits and assuming a ReLU activation function, the neuron output in an LQ-ANN is given by Equation (12). While both one-hot M-LIF SNNs with unit timestep (T=1) and LQ-ANNs share commonalities, they remain slightly distinct. As highlighted by Equations (11) and (12), they namely differ in their choices of firing threshold and their final output value ranges. They also differ in their training methods and abilities to extend to sequential processing (i.e., >1).

(12) $$o^{l}=\text{ReLU}\left(W^{l}{\tilde{o}}^{l-1}\right),\;{\tilde{o}}^{l-1}=\{\begin{array}{ll} \alpha^{l}2^{\text{clip}\left(\left\lfloor {\text{log}}_{2}\left(\frac{o^{l-1}}{\alpha^{l}}\right)\right\rceil ,1-2^{b},1\right)}, & \text{if}\;o^{l-1}\neq 0\\ 0, & \text{otherwise} \end{array}$$

## IV. ENERGY CONSUMPTION ESTIMATION

We evaluate the inference energy of our approach based on the approach in [15] and [16]. In conventional SNNs, 32-bit floating-point (FP32) additions replace the FP32 multiply-and-accumulates (MACs) of ANNs except in the first layer which uses direct encoded inputs. For one-hot M-LIF SNNs (*ω* = 1), inputs (outputs) are restricted to powers of 2, and multiplying by a power of 2 corresponds to adjusting the 8-bit integer (INT8) exponent of the FP32 multiplicand (see Appendix B). Therefore, scaling the intermediate FP32 membrane potential by 2*^s^* during integration in Equation (7) corresponds to increasing or decreasing its exponent in the INT8 format. According to [12], an INT8 addition consumes 30× less energy than a FP32 addition, hence the overhead of scaling in M-LIF SNNs is negligible and FP32 additions dominate the energy consumption of one-hot M-LIF SNNs. It is important to note that due to the one-hot constraint, the overall spiking rate (and consequently, the number of additions) per layer per timestep in one-hot M-LIF SNNs is not necessarily higher than that of conventional SNNs, even though one-hot M-LIF SNNs have multiple spiking lanes per neuron. This gives one-hot M-LIF SNNs the opportunity to learn more within a single timestep without increasing the computational complexity and energy compared to conventional SNNs.

It is also known that memory access energy can be significantly higher than compute energy [12], [41] and that a proportion of the number of memory accesses scales linearly with the number of timesteps in SNNs [16], [38]. However, estimating memory energy improvements would depend on hardware architecture and system configuration. Therefore, as noted in [16], we are restricting our attention to the computational energy benefits, *δ* defined in Equation (13) [16], of one-hot M-LIF SNNs and conventional SNNs over ANNs. As a result, we consider *δ* to be an optimistic energy gain estimate when *T* > 1. Note that when *T* = 1, memory requirements are identical for both SNNs and full-precision ANNs. When an iso-architecture ANN does not exist as in the case of spike-driven transformers [15] due to unique mechanisms such as spike-driven attention, we compare directly using the computational energy *E*.

As depicted in Figure 1, the one-hot M-LIF neuron model does not introduce any additional neurons to the existing set of neurons in iso-architecture SNNs and ANNs. As a result, ANNs and unit-timestep SNNs (both one-hot M-LIF and traditional LIF) have the same computational complexity. The computational complexity of multi-timestep SNNs, both one-hot M-LIF and traditional LIF, scales linearly with the number of timesteps as the inference process is repeated for T timesteps. Note however, that multi-timestep and unit-timestep SNNs, both one-hot M-LIF and traditional LIF, exhibit high activation sparsity $r_{l}^{t}$ per layer at each timestep enabling linear reduction in computational complexity. In Equation (13), the computational complexities #ANN_ops,*l*_ and #SNN_ops,*l*_ of layers in ANNs and SNNs are given in Equations (14) and (15) for convolution layers and in Equations (16) and (17) for linear layers, respectively. Based on [12], we set the relative MAC and addition energy to 4.6pJ and 0.9pJ, respectively. $C_{l}^{i},C_{l}^{o},k_{l},H_{l}$, and $W_{l}$ denote the number of input channels, number of output channels, kernel size, feature map height, and feature map width, respectively. These computational complexities are employed along with Equation 13 calculate the computational energy expenditure reported in Tables 1, 2, and 4 throughout Section V. Finally, note that in one-hot M-LIF SNNs, only a single spike lane fires per timestep as explained in Section III-B. The spiking activity is already accounted for in the term $r_{l}^{t}$. Therefore, the computational complexity does not explicitly depend on the number of spike lanes per timestep.

(13) $$\delta =\frac{E_{\text{ANN}}}{E_{\text{SNN}}}=\frac{\sum_{l=1}^{L}\#{\text{ANN}}_{\text{ops},l}\times 4.6}{\#{\text{SNN}}_{\text{ops},1}\times 4.6+\sum_{l=2}^{L}\#{\text{SNN}}_{\text{ops},l}\times 0.9}$$

(14) $$\#{\text{ANN}}_{\text{ops},l_{\mathrm{conv}}}=H_{l_{\mathrm{conv}}}\times W_{l_{\mathrm{conv}}}\times C_{l_{\mathrm{conv}}}^{i}\times C_{l_{\mathrm{conv}}}^{o}\times k_{l_{\mathrm{conv}}}^{2}$$

(15) $$\#{\text{SNN}}_{\text{ops},l_{\text{conv}}}=\overset{T}{\underset{t=1}{\sum }}r_{l}^{t}\times \#{\text{ANN}}_{\text{ops},l_{\text{conv}}}$$

(16) $$\#{\text{ANN}}_{\text{ops},l_{\text{linear}}}=C_{l_{\text{linear}}}^{i}\times C_{l_{\text{linear}}}^{o}$$

(17) $$\#{\text{SNN}}_{\text{ops},l_{\text{linear}}}=\overset{T}{\underset{t=1}{\sum }}r_{l}^{t}\times \#{\text{SNN}}_{\text{ops},l_{\text{linear}}}$$

## V. EXPERIMENTS AND RESULTS

We validate our one-hot M-LIF model and compare the performance and inference energy of our one-hot M-LIF SNNs with existing SNN works on both static and dynamic image classification tasks. Our proposed neuron model can be integrated into existing SNN training methodologies. We compare against the hybrid training methods [13], [16] for static tasks (Section V-A) and the temporal efficient training method [14] for dynamic vision tasks (Section V-B). We also evaluate the impact of one-hot M-LIF on more complex SNN-based high performance models such as the spike-driven transformer [15]. As in prior works, we employ direct input encoding for static tasks such that the input layer is fed with full-precision pixels. We also fix all layers to use the same number of (input) output spike lanes, *S*, as this reduces the number of hyperparameters. While certain hyperparameter, dataset, network architecture are highlighted in the following section, a detailed account of experimental settings such as optimizers, learning rates and schedules, number of epochs, number, and type of layers in different networks, is provided in Appendix C. The source code is available at: https://github.com/pabillam/one-hot-mlif-snn.

### A. STATIC IMAGE CLASSIFICATION

#### 1) IMPLEMENTATION DETAILS

We apply hybrid direct training as described in [13] and [16] to evaluate the accuracy of our approach on CIFAR10, CIFAR100, and ImageNet using VGG16 and ResNet20. We train an ANN with batch-norm [42] and subsequently fuse the batch-norm parameters with the weights of the corresponding layer. We then copy the weights of the pre-trained ANN to an iso-architecture one-hot M-LIF SNN and use the 90-th percentile of the input activation distribution as each layer’s threshold *θ^l^*. The SNN is then trained using BPTT but without temporal pruning. For spike-driven transformer, we evaluate our approach on CIFAR10, CIFAR100, and ImageNet using Transformer-2-512 and Transformer-8-512 by replacing all LIF neurons with one-hot M-LIF neurons while using the same training methodology as in [15]. Note that Transformer-*L*-*D* represents a model with *L* encoder blocks and *D* channels. These networks are trained from scratch using BPTT without any pre-trained ANN initialization or batch norm fusion. Supplemental network architecture details and hyperparameters are discussed in Appendix C.

#### 2) COMPARISON WITH SNNS

Table 1 compares the accuracy and inference energy of one-hot M-LIF SNNs with iso-architecture conventional SNNs. While our approach offers comparable or slightly lower energy benefits across most benchmarks, it consistently matches or exceeds conventional SNNs in accuracy. The one-hot constraint ensures energy usage comparable to conventional SNNs in the worst-case (all neurons fire at each timestep) despite each M-LIF neuron having multiple spiking lanes, discovering new accuracy-energy tradeoff points. Prior work achieved 69% accuracy with a unit timestep on ImageNet using VGG16, while we reached 71.05% with *S* = 3 spike lanes. For spike-driven transformers, M-LIF SNNs boost accuracy by up to 3% on ImageNet compared to LIF counterparts, consuming slightly more energy for a given *T* but achieving better tradeoffs. This is the case of (*T* = 1, *S* = 3) one-hot M-LIF spike-driven transformer, which achieves comparable or better accuracy to (*T* = 4) LIF on CIFAR100 and ImageNet with 4× less memory access energy (which can dominate overall energy as discussed in Section IV and in Appendix E) due to multi-timestep processing.

#### 3) COMPARISON WITH LQ-ANNS

As discussed in Section III-B, LQ-ANNs and unit timestep (*T* = 1) M-LIF SNNs remain distinct while both perform inference using a single timestep. Here, we compare the accuracy and inference energy of *b*-bit LQ-ANNs and M-LIF-based SNNs using *S* spike lanes as shown in Table 2. We observe that M-LIF SNNs perform on par or better than LQ-ANNs in terms of accuracy and inference energy. For CIFAR10, we observe similar accuracy and energy benefits to LQ-ANNs. On the other hand, for CIFAR100, we note that one-hot M-LIF SNNs are up to 54% more energy efficient than LQ-ANNs with comparable accuracy. Finally, our approach scales much better on a large challenging dataset such as ImageNet yielding > 3% accuracy improvement. This gain can be primarily attributed to threshold parameter learning for SNNs.

#### 4) COMPARISON WITH QUANTIZED-ACTIVATION SNNS

As discussed in Section II-E, prior works have explored the interplay between activation bit-width and timesteps in order to improve SNN accuracy. In this section, we perform a quantitative comparison provided in Table 3 with reported accuracies and energy estimates (when available) of burst-spike and multi-threshold methods on all evaluated static image classification datasets. Compared to burst-spike SNNs [36], [37], our one-hot M-LIF SNNs significantly reduce the number of timesteps (8 – 32×) while achieving better accuracy (up to > 4%) on more challenging datasets such as ImageNet. Compared to multi-threshold SNNs [22], [23] our one-hot M-LIF SNNs achieve better accuracy (up to > 4%) while significantly reducing computational energy. This is due to the introduction of uniform activation quantization in multi-threshold SNNs which breaks the multiplication-free property of SNNs.

### B. DYNAMIC IMAGE CLASSIFICATION

#### 1) IMPLEMENTATION DETAILS

For the dynamic image classification task where SNN accuracy is generally superior than that of ANNs, we apply temporal efficient training similar to [14] using our one-hot M-LIF neuron. Here, we train from scratch using BPTT without any pre-trained ANN initialization or batch norm fusion. We perform experiments on DVS-CIFAR10 [24] (converted from CIFAR10) which is one of the most challenging mainstream dynamic vision datasets. It has 10k images with size 128×128. Following prior works, we reduce the spatial resolution to 48 × 48, and split the dataset into 9k training and 1k test images [43]. We also apply data augmentation techniques such as random horizontal flip and random roll within 5 pixels [44]. For all experiments, we use the VGGSNN architecture [14] using 300 epochs, the Adam optimizer with learning rate λ = 0.001 and a cosine annealing scheduler with 0 decay. Details regarding the VGGSNN architecture [14] along with firing thresholds, membrane leakage, and surrogate gradient settings are included in Appendix C.

#### 2) MULTI-LEVEL INPUT LAYER ENCODING

**FIGURE 4.**
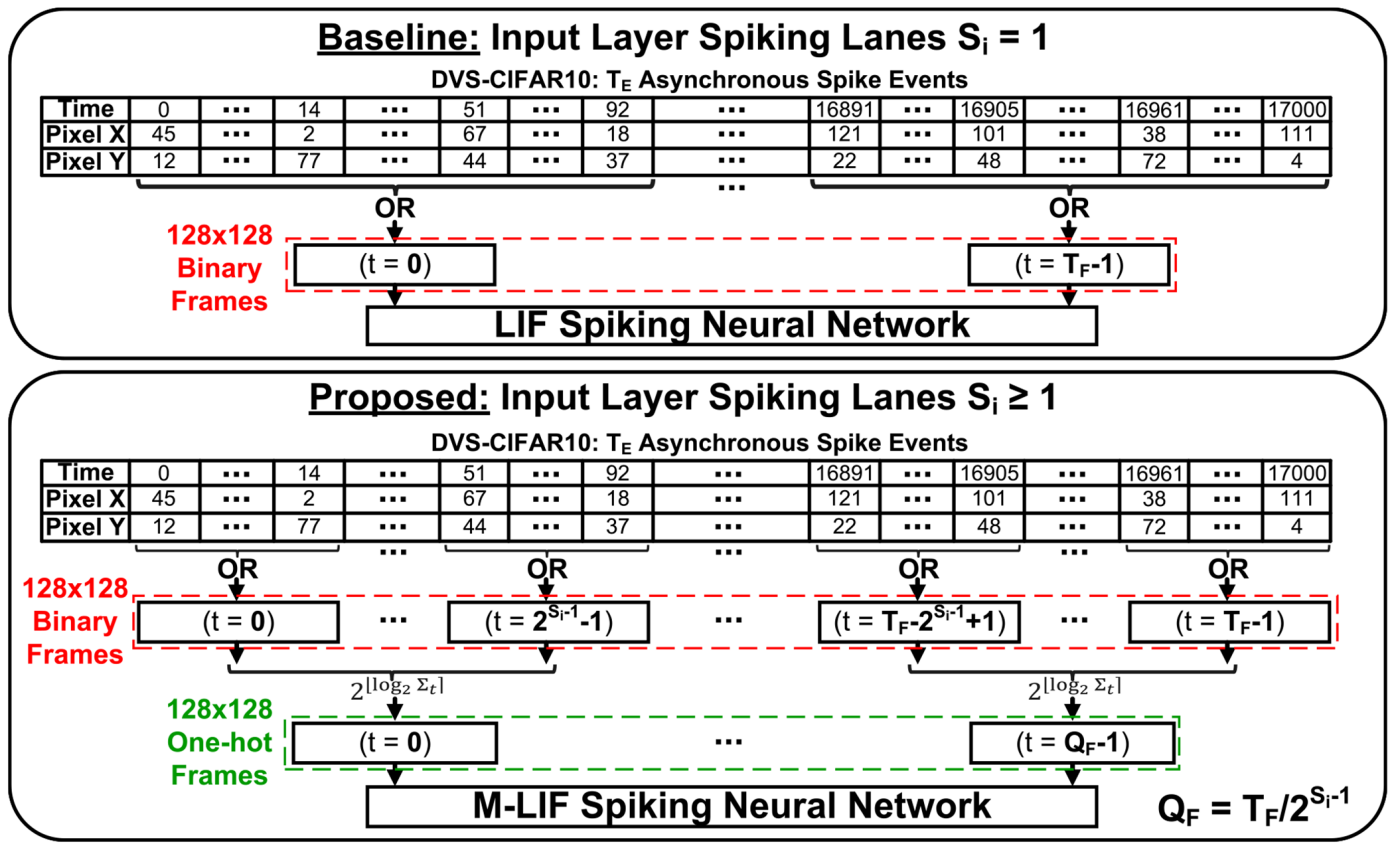
Workflow of multi-level input layer encoding for dynamic vision tasks.

For DVS-CIFAR10, direct input encoding is not applicable as the dataset consists of events recorded using a dynamic vision sensor. The adopted methodology described in [43] to prepare the data for SNN training is to split and convert the stream of $T_{E}$ events into $T_{F}$ binary frames as depicted in Figure 4 (top). In [14], a VGGSNN is trained with $T_{F}=10$ and a top-1 accuracy of 83.17%. However, M-LIF SNNs are not limited to single spike lanes at the input layer. Therefore, we allow $T_{F}\neq 10$ and incorporate an additional data preparation step to perform multi-level input layer encoding as depicted in Figure 4 (bottom). After obtaining the $T_{F}$ binary frames, we combine every $2^{S_{i}-1}$ consecutive frames into a one-hot frame resulting in $Q_{F}=T_{F}/2^{S_{i}-1}$ frames of one-hot $S_{i}$ spike lanes. This enables M-LIF SNNs to limit accuracy degradation after reducing $Q_{F}$ below the number of timesteps $T_{F}$.

#### 3) COMPARISON WITH SNNS

We compare against existing works on DVS-CIFAR10 in Table 4. The compute energy is calculated using $E_{\text{SNN}}=\sum_{l=1}^{L}\;\#{SNN}_{ops,l}\times 0.9pJ$, where $\#{SNN}_{ops,l}$ is defined in Section IV and 0.9 pJ is the energy of addition [12]. We achieve an accuracy of 84.7% using 10 timesteps and 4 spike lanes per neuron. This is also the first SNN work to achieve 82.5% accuracy on DVS-CIFAR10 using 3 timesteps and 4 spike lanes compared to the best prior work [19] which can only achieve 82.6% using 4.5 timesteps and 77.6% using 2.5 timesteps. These improvements in accuracy stem primarily from introducing the S dimension. By reducing $Q_{F}$, not only do we improve the computational energy by 3.45×, we also reduce membrane potential and spike memory access energy which scales linearly with timesteps and can be significantly higher than compute energy [16]. Table 4 also shows the impact of scaling $Q_{F}$ and S on accuracy. By increasing S for a fixed $Q_{F}$, we are able to recover accuracy degradation unlike prior works which are limited by solely scaling $T_{F}$. By increasing S and $Q_{F}$, we can scale the accuracy to even higher than conventional SNNs.

## VI. FUTURE DIRECTIONS

In this work, we introduce and evaluate a novel algorithmic optimization to existing SNNs, namely the one-hot M-LIF neuron model. We distinguish this approach from other multi-threshold and burst-spike SNNs as well as unit-timestep SNNs and log-quantized ANNs, and demonstrate that one-hot M-LIF SNNs provide a better accuracy tradeoff in low-latency (unit-timestep) regimes on a wide range of network architectures for both static and dynamic image classification tasks. However, several areas remain unexplored.

Expanding experiments beyond image-based datasets (CIFAR10, CIFAR100, ImageNet, DVS-CIFAR10) to include speech and text tasks, such as in [51] and [52], would enhance generalizability. Robustness to noise and data distribution shifts is another critical aspect for real-world deployment. Integrating techniques from works solely focused on training robust SNNs such as [53] and [54] into one-hot M-LIF SNN training could address this gap. Many prior works have also investigated the topic of hardware architectures and dataflow optimizations for existing SNN algorithms on ASIC or FPGA platforms [55], [56], [57], [58]. For example, [55] discusses the feasibility of leveraging systolic arrays to perform SNN inference using a small number of timesteps. The one-hot M-LIF neuron model was designed to be adaptable to many existing SNN hardware accelerator architectures. Our work highlights the potential advantages of one-hot M-LIF SNNs over traditional SNNs through energy modeling. Future work could investigate the complete implementation and execution of one-hot M-LIF SNNs on ASIC or FPGA which would then further validate its hardware advantages. This aspect is also critical for practical deployment of one-hot M-LIF SNNs in low-power and real-time scenarios such as in edge computing, autonomous driving, and IoT systems.

## VII. CONCLUSION

SNNs hold promise as an energy-efficient alternative to traditional ANNs. However, achieving an optimal balance in the accuracy-energy tradeoff by adjusting latency remains a significant challenge for widespread deployment. To that end, we introduce the dimension of spike lanes to conventional SNNs using a novel M-LIF neuron model without latency and computational complexity overhead. The proposed model represents the inputs and outputs of hidden layers as a set of one-hot binary-weighted spike lanes. Using our one-hot M-LIF neuron model, we are able to find new and better tradeoff points for both static and dynamic vision tasks. In particular, our one-hot M-LIF-based SNNs achieve a top-1 accuracy of 71.05% on ImageNet using VGG16 and enhance the computational efficiency by 20×. One-hot M-LIF neurons also improve the accuracy-latency tradeoff for advanced network architectures such as spike-driven transformers (> 3% higher accuracy and 4× fewer timesteps on ImageNet). For dynamic vision tasks, such as image classification using dynamic vision sensor data, our one-hot M-LIF SNNs retain higher accuracy (82.5%) when scaling down to fewer timesteps (3) on CIFAR10-DVS thus providing better energy efficiency.

## Figures and Tables

**TABLE 1.** Iso-architecture comparison with SNNs for static image classification on CIFAR and ImageNet.

Dataset	Architecture	Method	*S*	*T*	Accuracy (%)	*δ*	Comp Energy (*μ*J)
CIFAR10	ResNet20	ANN*	-	1	94.31	1.0	9.97E+02

		DIET-SNN [13]	1	5	91.78	6.3	1.58E+02
		Temporal Pruning [16]	1	1	91.10	16.32	6.11E+01
		**1-hot M-LIF (ours)**	**3**	**1**	**93.19**	**18.10**	**5.55E+01**

	VGG16	ANN*	-	1	94.43	1.0	1.56E+03

		DIET-SNN [13]	1	5	92.70	12.4	1.26E+02
		Temporal Pruning [16]	1	1	93.05	**33.0**	**4.72E+01**
		BANN [38]	1	1	**93.44**	25.08	6.22E+01
		**1-hot M-LIF (ours)**	3	1	93.34	29.73	5.24E+01

	Transformer-2-512	Spike-Driven Transformer [15]*	1	4	95.6	-	4.60E+02
		Spike-Driven Transformer [15]*	1	2	94.7	-	2.80E+02
		Spike-Driven Transformer [15]*	1	1	94.5	-	**1.92E+02**

		**1-hot M-LIF (ours)**	3	4	**95.9**	-	1.47E+03
		**1-hot M-LIF (ours)**	3	2	95.5	-	4.84E+02
		**1-hot M-LIF (ours)**	3	1	95.4	-	2.59E+02

CIFAR100	ResNet20	ANN*	-	1	67.10	1.0	9.97E+02

		DIET-SNN [13]	1	5	**64.07**	6.6	1.51E+02
		Temporal Pruning [16]	1	1	63.30	**15.35**	**6.50E+01**
		**1-hot M-LIF (ours)**	3	1	63.80	14.05	7.10E+01

	VGG16	ANN*	-	1	74.50	1.0	1.56E+03

		DIET-SNN [13]	1	5	69.97	12.1	1.29E+02
		Temporal Pruning [16]	1	1	70.15	**29.24**	**5.34E+01**
		**1-hot M-LIF (ours)**	3	1	**72.59**	23.63	6.60E+01

	Transformer-2-512	Spike-Driven Transformer [15]*	1	4	78.4	-	5.87E+02
		Spike-Driven Transformer [15]*	1	2	76.6	-	**1.90E+02**
		Spike-Driven Transformer [15]*	1	1	75.8	-	2.21E+02

		**1-hot M-LIF (ours)**	3	4	**78.9**	-	1.68E+03
		**1-hot M-LIF (ours)**	3	2	78.3	-	8.20E+02
		**1-hot M-LIF (ours)**	3	1	78.2	-	4.78E+02

ImageNet	VGG16	ANN*	-	1	72.56	1.0	7.12E+04

		DIET-SNN [13]	1	5	69.00	11.7	6.09E+03
		Temporal Pruning [16]	1	1	69.00	**24.61**	**2.89E+03**
		BANN [38]	1	1	68.00	20.94	3.40E+03
		**1-hot M-LIF (ours)**	3	1	**71.05**	20.20	3.37E+03

	Transformer-8-512	Spike-Driven Transformer [15]	1	1	71.68	-	**1.13E+03**
		Spike-Driven Transformer [15]	1	4	74.57	-	4.50E+03

		**1-hot M-LIF (ours)**	3	1	**75.33**	-	3.64E+03

* denotes self-implementation results.

**TABLE 2.** Iso-architecture comparison with LQ-ANNs for static image classification on CIFAR and ImageNet.

Dataset	Architecture	Method	*S*	*T*	*b*	Accuracy (%)	*δ*	Comp Energy (*μ*J)
CIFAR10	ResNet20	LQ-ANN*	-	1	2	93.03	**21.60**	**4.61E+01**
			-	1	3	**93.59**	12.02	8.29E+01

		**1-hot M-LIF (ours)**	2	1	-	92.61	20.78	4.80E+01
			3	1	-	93.19	18.10	5.51E+01

CIFAR100	VGG16	LQ-ANN*	-	1	2	71.24	25.28	6.17E+01
			-	1	3	**72.87**	15.29	1.02E+02

		**1-hot M-LIF (ours)**	2	1	-	71.32	**27.30**	**4.88E+01**
			3	1	-	72.59	23.63	5.66E+01

ImageNet	VGG16	LQ-ANN*	-	1	3	62.97	16.95	4.20E+03
			-	1	4	67.85	14.18	5.02E+03

		**1-hot M-LIF (ours)**	3	1	-	**71.05**	**20.20**	**3.37E+03**

* denotes self implementation results.

**TABLE 3.** Comparison with quantized-activation SNNs for static image classification on CIFAR and ImageNet.

Dataset	Method	Architecture	*T*	Accuracy (%)	Comp Energy (mJ)
CTFAR10	MLF(*K* = 3) [23]	Spiking DS-ResNet	4	94.25	-
	Cascade-MT (4-bit) [22]	VGG9	1	95.17	-
	Burst-Spike+LIPooling [37]	VGG16	32	95.58	-
	**1-hot M-LIF** (*S* = 3) **(ours)**	**Transformer-2-512**	**1**	**95.4**	**2.59E-01**

CIFAR100	Parallel-MT (2-bit) [22]	VGG9	1	73.89	4.40E+02
	Cascade-MT (2-bit) [22]	VGG9	1	74.80	7.40E+02
	Burst-Spike+LIPooling [37]	VGG16	32	74.98	-
	**1-hot M-LIF (S=3) (ours)**	**Transformer-2-512**	**1**	**78.2**	**4.78E-01**

ImageNet	Parallel-MT (2-bit) [22]	ResNet-34	1	69.32	-
	Cascade-MT (4-bit) [22]	ResNet-34	1	72.17	-
	Burst-Spike (*ψ* = 4) [36]	VGG16	8	70.61	-
	Burst-Spike+LIPooling [37]	VGG16	32	70.18	-
	**1-hot M-LIF** (*S* = 3) **(ours)**	**Transformer-8-512**	**1**	**75.33**	**3.64E+00**

**TABLE 4.** Comparison with prior works for dynamic image classification on DVS-CIFAR10.

Method	Architecture	*S*	*T_F_*	*Q_F_*	Accuracy (%)	Comp Energy (*μ*J)
STBP-tdBN [45]	ResNet-19	1	10	-	67.8	-
Streaming Rollout [46]	DenseNet	1	10	-	66.8	-
Conv3D [47]	LIAF-Net	1	10	-	71.70	-
LIAF [47]	LIAF-Net	1	10	-	70.40	-
Dspike [48]	ResNet-18	1	10	-	75.4	-
RecDis-SNN [49]	ResNet-19	1	10	-	72.4	-
Spike-driven Transformer^†^ [15]	Transformer-2-256	1	16	-	80.0	-
Burst-Spike (*ψ* = 8) [36]	ResNet-18	1	8	-	80.41	-
MLF (*K* = 3) [23]	Spiking DS-ResNet	1	10	-	70.36	-
PLIF [50]	VGGSNN	1	20	-	74.8	-
SEENN-II^†^ [19]	VGGSNN	1	4.5	-	82.6	-
SEENN-I^†^ [19]	VGGSNN	1	**2.5**	-	**77.6**	-
TET [14]	VGGSNN	1	10	-	77.3	-
TET*^†^ [14]	VGGSNN	1	**10**	-	**83.1**	3.8E+02
TET*^†^ [14]	VGGSNN	1	5	-	78.0	1.9E+02
TET*^†^ [14]	VGGSNN	1	**3**	-	**74.7**	**1.2E+02**

**1-hot M-LIF**† **(ours)**	VGGSNN	4	-	**10**	**84.7**	**3.5E+02**
**1-hot M-LIF**† **(ours)**	VGGSNN	3	-	10	84.3	3.4E+02
**1-hot M-LIF**† **(ours)**	VGGSNN	4	-	5	83.3	1.8E+02
**1-hot M-LIF**† **(ours)**	VGGSNN	3	-	5	83.0	1.7E+02
**1-hot M-LIF**† **(ours)**	VGGSNN	4	-	**3**	**82.5**	**1.1E+02**
**1-hot M-LIF**† **(ours)**	VGGSNN	3	-	3	79.8	9.0E+01

† denotes data augmentation.

* denotes self-implementation results.

**FIGURE 5.**
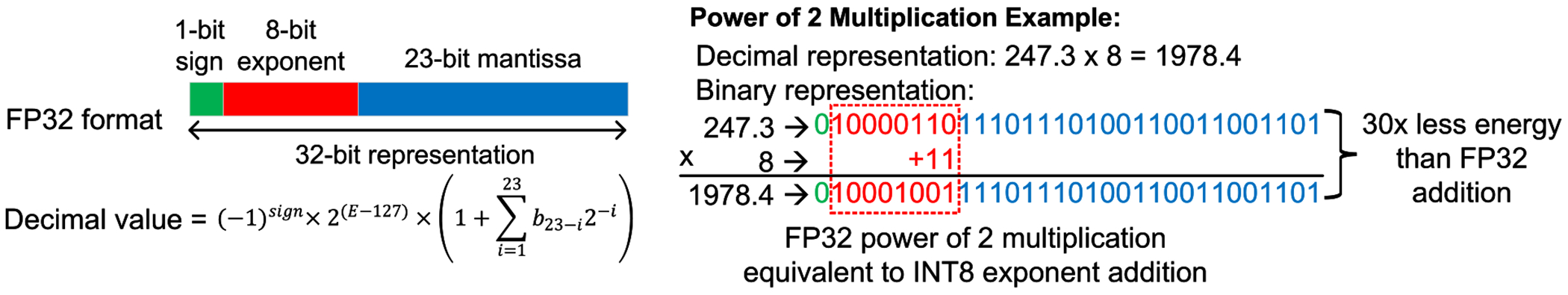
32-bit floating-point format and power of two multiplication details where *b_i_* is the *i*-th bit, *sign* is the most significant bit, and *E* is the 8-bit exponent.

**FIGURE 6.**
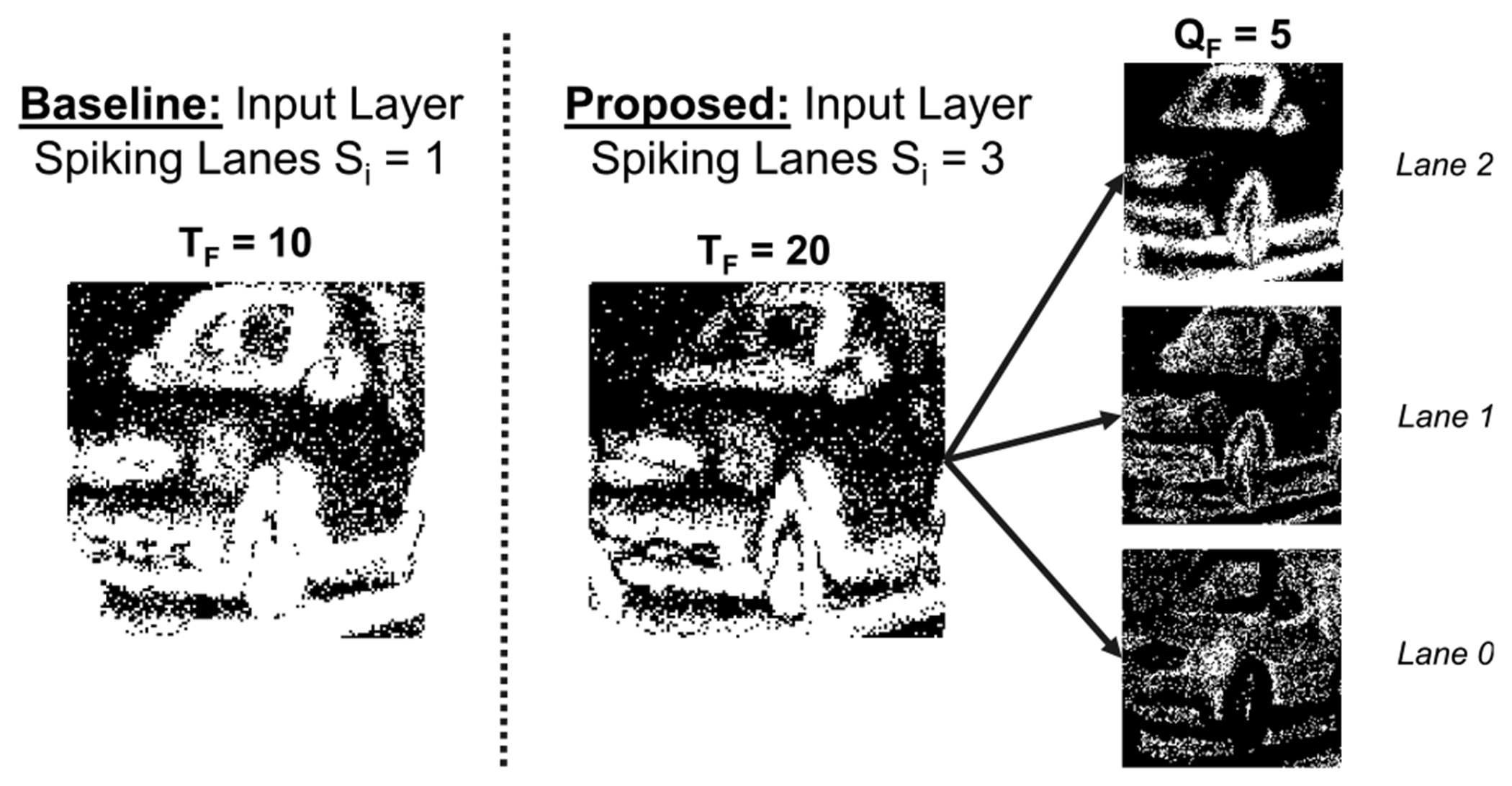
Example image in DVS-CIFAR10 using baseline input layer encoding with *T_F_* = 10 binary frames and *S_i_* = 1 spike lane (left) and the proposed multi-level input layer encoding with *Q_F_* = 5 one-hot frames and *S_i_* = 3 spike lanes (right).

**FIGURE 7.**
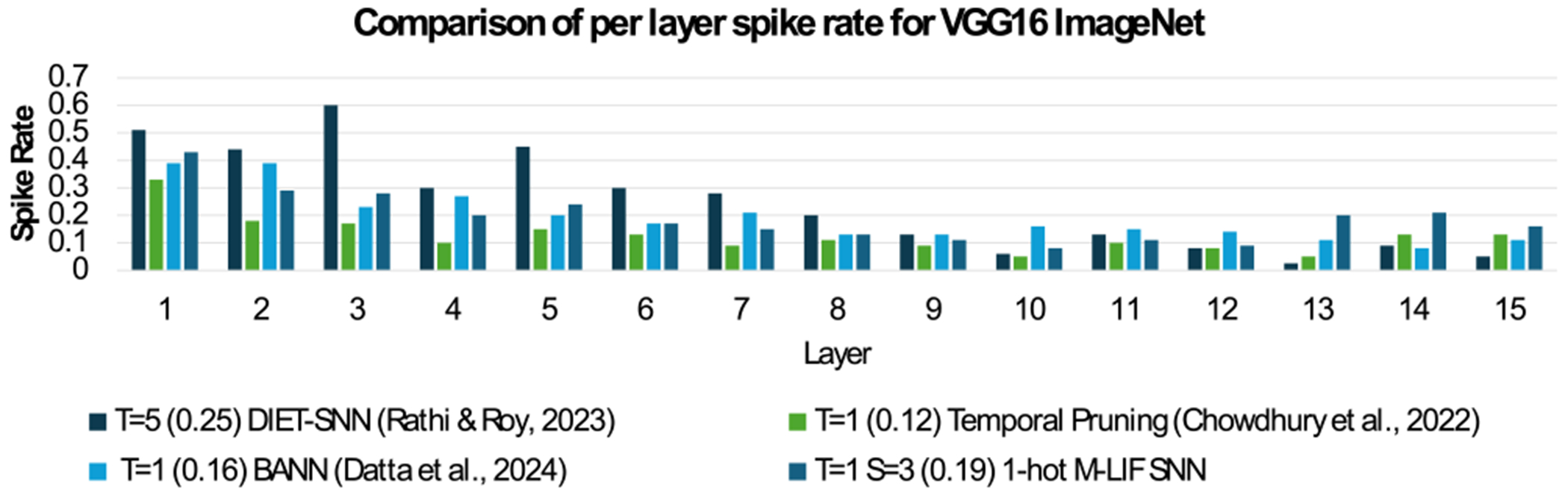
Comparison of per-layer spike rates for VGG16 on ImageNet.

## APPENDIX A SURROGATE GRADIENT

Adapting the derivative of sigmoid function *σ*′ to one-hot M-LIF is performed in a similar fashion to the triangular surrogate gradient described in the main text. The surrogate gradient becomes the difference of *σ*′ sub-gradients, one for each of the rising and falling window edges of the firing function, as shown in Equation (18).

$$z^{l}(s)=\sigma \left(\alpha \left(\frac{u^{l}[t]}{2^{s}\theta^{l}}-1\right)\right)$$

$$q^{l}(s)=\frac{\alpha }{2^{s}\theta^{l}}\left(1-z^{l}(s)\right)z^{l}(s)$$

$$\frac{\partial o^{l}[t]}{\partial u^{l}[t]}=\overset{S_{o}-1}{\underset{s=0}{\sum }}2^{s}\frac{\partial o_{s}^{l}[t]}{\partial u^{l}[t]}$$

(18) $$\frac{\partial o_{s}^{l}[t]}{\partial u^{l}[t]}=\{\begin{array}{ll} \text{diag}\left(q^{l}(s)\right), & \text{if}\;s=S_{o}-1\\ \text{diag}\left(\overset{1}{\underset{a=0}{\sum }}{\left(-1\right)}^{a}q^{l}(s+a)\right), & \text{if}\;0\leq s<S_{o}-1 \end{array}$$

## APPENDIX B FP32 POWER OF TWO MULTIPLICATION

As discussed in the main text, both M-LIF SNNs and LIF SNNs perform FP32 additions during integration to calculate the membrane potential at each timestep. For M-LIF SNNs, FP32 weights are scaled by a power of 2 prior to integration. As illustrated in Figure 5 which provides details regarding the FP32 number format, power of two multiplication corresponds to adjusting the 8-bit integer exponent which requires 30× less energy than the FP32 addition [12]. Hence, the overhead is negligible and M-LIF SNNs are considered to leverage additions instead of multiplications like their LIF counterparts. Our proposed approach is designed to be adaptable to various hardware architectures, including custom ASICs or FPGAs, where the operation depicted in Figure 5 can indeed be implemented efficiently. It is also worth noting that modern commercial instruction set architectures, such as x86, include specific support for efficiently scaling floating-point numbers. For example, the x87 floating-point unit (FPU) provides specialized instructions like fscale [59] designed to scale floating-point numbers by powers of two, rather than performing a general floating-point multiplication. This indeed suggests that the operation described in Figure 5 aligns with existing commercial hardware capabilities.

## APPENDIX C EXPERIMENTAL DETAILS

### A. STATIC IMAGE CLASSIFICATION

#### 1) DATASETS

We employ the CIFAR datasets [60] which consist of 50k training and 10k test images. CIFAR10 contains 10 classes while CIFAR100 contains 100 classes. Standard data augmentation techniques are applied to training images such as padding by 4 pixels on each side, random horizontal flip and 32×32 crop by randomly sampling the padded image. During test, the original 32 × 32 images are used. We calculate the channel-wise mean and standard deviation of training images and use those values to normalize both training and test data. Contrary to other works such as [19], we do not apply augmentation techniques on CIFAR for CNN experiments such as Cutout [61] and AutoAugment [62] in order to faithfully compare with results in [16]. We employ the same augmentation techniques on CIFAR when comparing with results in [15]. We also employ the ImageNet dataset [1] which contains 1000 classes and consists of more than 1250k training and 50k test images. We employ the same augmentation techniques on ImageNet when comparing with results in [15] and [16].

#### 2) NETWORK ARCHITECTURES

The VGG16 and ResNet20 architectures adopted for static image classification are taken from [16]. Average pooling (2 × 2) is applied for all cases. The ResNet basic blocks use a 1 × 1 stride-2 convolutional layer shortcut path where the number of input and output channels are different. The architectures of each network are summarized below where BB denotes a basic block [63], D denotes a dropout layer (probability ANN: 0, SNN: 0.2), AP2 denotes a (2 × 2) average pooling layer, (/2) denotes stride-2, *o*C*k* denotes a (*k* × *k*) stride-1 convolutional layer with *o* output filters, *o*FC denotes a fully-connected layer with *o* output filers, and *n* denotes the number of classes.

VGG16: {64C3, D, 64C3, AP2, 128C3, D, 128C3, AP2, 256C3, D, 256C3, D, 256C3, AP2, 512C3, D, 512C3, D, 512C3, AP2, 512C3, D, 512C3, D, 512C3, D, 4096FC, 4096FC, *n*FC}.

ResNet20: {64C3, D, 64C3, D, 64C3, AP2, 64BB, 64BB, 128BB (/2), 128BB, 256BB (/2), 256BB, 512BB (/2), 512BB, *n*FC}.

The spike-driven transformer architectures adopted for static image classification are taken from [15]. Transformer-*L-D* networks include spiking patch splitting followed by *L* spike-driven encoder blocks and a linear classification head. The encoder blocks consist of a spike-driven self attention layer followed by two multi-layer perceptron layers. *D* refers to the number of channels in the input to the encoder blocks.

#### 3) TRAINING HYPERPARAMETERS

To train ANNs, we use a stochastic gradient descent optimizer with weight decay of 0.0005 and momentum of 0.9. We initialize ANN weights using He initialization [64]. We train the ResNet20 and VGG16 ANNs for CIFAR10 and CIFAR100 during 300 epochs with an initial learning rate of 0.01 that is divided by 10 at epochs 120, 180, and 240. We train VGG16 ANN for ImageNet during 90 epochs with an initial learning rate of 0.01 that is divided by 5 at epochs 40, 62, and 81. We also employ batch-norm during ANN training [42]. We use a pre-trained ANN to initialize the parameters of a corresponding LQ-ANN and perform training using an Adam optimizer with a weight decay of 0.0005 for 50 epochs and an initial learning rate of 0.0001. The learning rate is divided by 5 at epochs 20, 30, and 40.

For iso-architecture M-LIF SNN initialization from a pre-trained ANN, we fuse the batch-norm parameters with the corresponding layer’s parameters similar to [16]. For static image classification with VGG16 and ResNet20, we use a triangular surrogate gradient with *γ* = 0.3 and learn firing thresholds and membrane leakages during training as per [16]. The learning rate is shared among weights, firing thresholds, and membrane leakages unless otherwise noted. Upon initialization, we train M-LIF SNNs using an Adam optimizer with a weight decay of 0. ResNet20 M-LIF SNNs are trained on CIFAR10 for 150 epochs with an initial learning rate of 0.0001 that is divided by 5 at epochs 90, 120, and 135. ResNet20 M-LIF SNNs are trained on CIFAR100 for 600 epochs with a fixed learning rate of 0.01 for firing thresholds and an initial learning rate of 0.0002 that is divided by 2 at epochs 120, 240, 360, 450, and 540 for the remaining parameters. VGG16 M-LIF SNNs are trained on CIFAR10 for 200 epochs. During the first 10 epochs, the learning rate is increased linearly from 0.00001 to 0.0001 and subsequently divided by 5 at epochs 62 and 150. VGG16 M-LIF SNNs are trained on CIFAR100 for 200 epochs with a fixed learning rate of 0.001 for firing thresholds and an initial learning rate of 0.0001 that is divided by 5 at epochs 62 and 150 for the remaining parameters. VGG16 M-LIF SNNs are also trained on ImageNet for 50 epochs. During the first 5 epochs, the learning rate is increased linearly from 0.00001 to 0.0001 and subsequently divided by 5 at epochs 30 and 40.

**TABLE 5.** Ablation study varying the number of spike lanes for one-hot M-LIF VGGSNN on DVS-CIFAR10 with fixed timesteps *T* = 3 and *T* = 5.

*S*	Accuracy (%) for *T* = 3	Accuracy (%) for *T* = 5
1	74.7	78.0
2	78.6	81.5
3	79.8	83.0
4	82.5	83.3
5	82.5	82.9

For spike-driven transformers, we adopt the same hyperparameters as LIF spike-driven transformers in [15] when training their M-LIF counterparts for all experiments. We use a surrogate gradient based on the derivative of the sigmoid function with α=4. In order to make use of the same hyperparameter configuration, the outputs of M-LIF neurons are scaled to be in the range [0,1] but continue to be powers of 2 (*i.e.*, $\in 1,\frac{1}{2},\frac{1}{4},\frac{1}{8},\ldots$). Finally, similar to [15], we use a fixed threshold for all M-LIF neurons in spike-driven transformer experiments instead of learning the threshold as in our experiments on other architectures.

### B. DYNAMIC IMAGE CLASSIFICATION

#### 1) DATASET

We employ the DVS-CIFAR10 dataset with standard data augmentation techniques as discussed in Section V-B. Figure 6 illustrates an example training image of a car in DVS-CIFAR10 using the baseline input layer encoding with *T_F_* = 10 binary frames and *S_i_* = 1 spike lane (left) and the proposed multi-level input layer encoding with *Q_F_* = 5 one-hot frames and *S_i_* = 3 spike lanes (right).

#### 2) NETWORK ARCHITECTURE

The VGGSNN architecture employed was taken from [14] and is described as VGGSNN: {64C3, 128C3 (/2), 256C3, 256C3 (/2), 512C3, 512C3 (/2), 512C3, 512C3 (/2), 10FC}.

#### 3) TRAINING HYPERPARAMETERS

In addition to the training settings mentioned in Section 4.2 of the main paper, we use *γ* = 1.0 for all SNN surrogate gradient scaling and fix firing thresholds and membrane leakages to 1.0 and 0.5, respectively, for all layers as per [14].

## APPENDIX D ABLATION STUDIES

We conducted an ablation study where we varied the number of spike lanes *S* while keeping the number of timesteps *T* fixed at 3 for VGGSNN on DVS-CIFAR10. We provide the results in Table 5. Our findings indicate that as the number of spike lanes increases from *S* = 1 to *S* = 4, there is a significant improvement in accuracy, after which performance saturates. Additionally, when we increase the number of timesteps to *T* = 5 in Table 5, we see a similar performance saturation around *S* = 4 timesteps. We also note that the accuracy improvement due to spike lanes becomes less pronounced, but the overall accuracy ceiling improves. From these results, we conclude that both timesteps and spike lanes contribute to better model accuracy with the caveat that multi-timestep processing introduces significant energy overhead, particularly with respect to memory access as noted in Section IV.

**TABLE 6.** Impact of memory access energy for static image classification.

Model	Dataset	Method	*S*	*T*	Accuracy (%)	Comp Energy (*μ*J)	Mem Energy (*μ*J)	Total Energy (*μ*J)
VGG16	CIFAR10	ANN*	-	-	94.43	1.56E+03	1.89E+04	2.05E+04

		DIET-SNN [13]	1	5	92.7	1.26E+02	3.19E+03	3.32E+03
		Temporal Pruning [16]	1	1	93.05	4.72+01	2.15E+03	2.20E+03
		BANN [38]	1	1	93.44	6.22E+01	2.18E+03	2.24E+03
		**1-hot M-LIF (ours)**	**3**	**1**	**93.34**	**5.24E+01**	**2.18E+03**	**2.23E+03**

	CIFAR100	ANN*	-	-	74.5	1.56E+03	1.89E+04	2.05E+04

		DIET-SNN [13]	1	5	69.97	1.29E+02	3.01E+03	3.14E+03
		Temporal Pruning [16]	1	1	70.15	5.34E+01	2.16E+03	2.21E+03
		**1-hot M-LIF (ours)**	**3**	**1**	**72.59**	**6.60E+01**	**2.19E+03**	**2.26E+03**

	ImageNet	ANN*	-	-	72.56	7.12E+04	7.81E+05	8.52E+05

		DIET-SNN [13]	1	5	69.00	6.09E+03	5.88E+04	6.49E+04
		Temporal Pruning [16]	1	1	69.00	2.89E+03	1.55E+04	1.84E+04
		BANN [38]	1	1	68.00	3.40E+03	1.71E+04	2.05E+04
		**1-hot M-LIF (ours)**	**3**	**1**	**71.05**	**3.37E+03**	**2.21E+04**	**2.58E+04**

Transformer-2-512	CIFAR10	Spike-Driven Transformer [15]*	1	4	95.6	4.60E+02	2.10E+03	2.56E+03
		**1-hot M-LIF (ours)**	**3**	**1**	**95.4**	**2.59E+02**	**1.47E+03**	**1.73E+03**

	CIFAR100	Spike-Driven Transformer [15]*	1	4	78.4	5.87E+02	2.90E+03	3.49E+03
		**1-hot M-LIF (ours)**	**3**	**1**	**78.2**	**4.78E+02**	**2.65E+03**	**3.13E+03**

Transformer-8-512	ImageNet	Spike-Driven Transformer [15]	1	4	74.57	4.50E+03	4.31E+04	4.76E+04
		**1-hot M-LIF (ours)**	**3**	**1**	**75.33**	**3.64E+03**	**1.69E+04**	**2.05E+04**

* denotes self-implementation results.

## APPENDIX E MEMORY ACCESS ENERGY IMPACT

As discussed in Section IV, memory accesses are indeed dependent on the hardware architecture. However, defining a precise memory architecture requires detailed assumptions regarding weight, input, and partial output reuse. Given that a hardware architecture study is outside the scope of this paper, we include an analysis based on the memory energy model provided in [38, Appendix A.8.2]. The energy model equations $SNN_{ME}^{T>1}$ and $SNN_{ME}^{T=1}$ for multi-timestep and unit-timestep SNNs, respectively, are given in Equations (19) and (20).

(19) $${\text{SNN}}_{\text{ME}}^{T>1}=\overset{L}{\underset{l=1}{\sum }}C_{l}^{i}C_{l}^{o}k_{l}^{2}E_{\text{rd}}^{\text{wt}}+C_{1}^{i}C_{1}^{o}k_{l}^{2}H_{1}W_{1}E_{\text{rd}}^{\text{inp}}\;+\overset{T}{\underset{t=1}{\sum }}\left(\overset{L}{\underset{l=2}{\sum }}C_{l}^{i}C_{l}^{o}k_{l}^{2}H_{l}W_{l}r_{l}^{t}E_{\text{rd}}^{\text{sp}}\right)\;+\overset{T}{\underset{t=1}{\sum }}\left(\overset{L}{\underset{l=1}{\sum }}H_{l}W_{l}C_{l}^{o}\left(E_{\text{wr}}^{\text{sp}}+E_{\text{rd}}^{\text{mem}}+E_{\text{wr}}^{\text{mem}}\right)\right)$$

(20) $${\text{SNN}}_{\text{ME}}^{T=1}=\overset{L}{\underset{l=1}{\sum }}C_{l}^{i}C_{l}^{o}k_{l}^{2}E_{\text{rd}}^{\text{wt}}+C_{1}^{i}C_{1}^{o}k_{l}^{2}H_{1}W_{1}E_{\text{rd}}^{\text{inp}}\;+\overset{L}{\underset{l=2}{\sum }}C_{l}^{i}C_{l}^{o}k_{l}^{2}H_{l}W_{l}r_{l}^{t}E_{\text{rd}}^{\text{sp}}+\overset{L}{\underset{l=1}{\sum }}H_{l}W_{l}C_{l}^{o}E_{\text{wr}}^{\text{sp}}$$

$C_{l}^{i},C_{l}^{o},k_{l},H_{l},W_{l},E_{\mathrm{rd}}^{\mathrm{wt}},E_{\mathrm{rd}}^{\mathrm{inp}},E_{\mathrm{rd}}^{\mathrm{sp}},E_{\mathrm{wr}}^{\mathrm{sp}},E_{\mathrm{rd}}^{\mathrm{mem}},E_{\mathrm{wr}}^{\mathrm{mem}}$, and $r_{l}^{t}$ denote the number of input channels, number of output channels, kernel size, feature map height, feature map width, read energy of weights from memory, read energy of full-precision first layer inputs from memory, read energy of spike inputs from memory, write energy of spike outputs to memory, read energy of full-precision membrane potentials from memory, write energy of full-precision membrane potentials to memory, and the activation sparsity of layer l at timestep t, respectively.

We evaluate these equations using the same 45 nm CMOS technology for energy modeling [12], allowing for consistent energy comparisons, with $E_{\mathrm{wr}}^{\mathrm{mem}}=E_{\mathrm{rd}}^{\mathrm{mem}}=E_{\mathrm{rd}}^{\mathrm{wt}}=E_{\mathrm{rd}}^{\mathrm{inp}}=50pJ$ and $E_{\mathrm{rd}}^{\mathrm{sp}}=1.56pJ$. For one-hot M-LIF SNNs, input and output spikes are now replaced by power-of-two activations using ${log}_{2}\left(S_{o}^{l}+1\right)$ bits, and read/write energies of spike inputs/outputs to/from memory are scaled by ${log}_{2}\left(S_{o}^{l}+1\right)$. Our ablation studies in Appendix D show an accuracy performance saturation at around $S_{o}^{l}=4$, which translates to negligible 1 to 2 bit overhead considering the high activation sparsity and the much larger full-precision membrane potential read/write energies. Therefore, one-hot M-LIF SNNs do not introduce significant additional memory overhead compared to traditional SNNs using the same number of timesteps. Of course, memory overhead is not negligible in the case of multiple-timestep SNNs compared to ANNs (which effectively use a single timestep). In the unit-timestep case, we have the same number of memory accesses but benefit from smaller activation bit-widths.

With this model, we can estimate the impact of memory energy. However, the model factors in per layer activation sparsity, something which is not thoroughly provided to us for all prior works [13], [16], [38] due to lack of model checkpoints publicly available. We include a comparison for a model whose spike rates were provided across multiple prior works (VGG16 on CIFAR10, CIFAR100, and ImageNet; Transformer-8-512 on ImageNet). We also make use of self-implementation results using iso-accuracy one-hot M-LIF and traditional LIF spike-driven Transformer-2-512 on CIFAR10 and CIFAR100.

Table 6 illustrates the impact of memory access energy on the overall energy comparison. For VGG16 on ImageNet, one-hot M-LIF SNN consumes 33× lower energy than ANNs, while maintaining higher accuracy (up to > 3%) than prior unit-timestep SNN works. We also observe that multi-timestep processing introduces a noticeable energy overhead. As indicated in Equation (19), the model assumes a small number of timesteps is employed so as to reuse weights across all timestep calculations. As the number of timesteps increases, this assumption no longer holds as redundant weight accesses are performed. However, the memory access energy for membrane potentials and spikes scales linearly with the number of timesteps. For small feature map dimensions such as in CIFAR, the impact of this term is manageable. This observation matches the results shown in Table 6 for CIFAR. However, image dimensions in CIFAR (32 × 32) are not considered realistic for most real-world image classification tasks. Increasing the per layer feature map dimensions *H_l_* and *W_l_* to something more realistic as in the case of ImageNet (224 × 224), the memory access energy for membrane potentials and spikes starts to dominate the overall memory energy consumption for larger number of timesteps. In fact, for VGG16 on ImageNet, we achieve higher accuracy compared to (*T* = 5) [13] while consuming 2.5× less total energy, the majority of which stems from the memory energy overhead of multi-timestep (*T* = 5) membrane potential access. Figure 7 provides per layer spike rates for VGG16 ImageNet of the models compared in Table 6. Similarly, for Transformer-8-512 on ImageNet, we achieve higher accuracy compared to (*T* = 4) [15] while consuming 2.3× less total energy, the majority of which stems from the memory energy overhead of multi-timestep (*T* = 4) membrane potential access.

## References

[1] Krizhevsky A, Sutskever I, and Hinton GE, “ImageNet classification with deep convolutional neural networks,” in Proc. Adv. Neural Inf. Process. Syst, vol. 60, Pereira F, Burges C, Bottou L, and Weinberger K, Eds., May 2017, pp. 84–90. [Online]. Available: https://proceedings.neurips.cc/paper_files/paper/2012/file/c399862d3b9d6b76c8436e924a68c45b-Paper.pdf

[2] Redmon J, Divvala S, Girshick R, and Farhadi A, “You only look once: Unified, real-time object detection,” in Proc. IEEE Conf. Comput. Vis. Pattern Recognit. (CVPR), Jun. 2016, pp. 779–788.

[3] Maass W, “Networks of spiking neurons: The third generation of neural network models,” Neural Netw., vol. 10, no. 9, pp. 1659–1671, Dec. 1997.

[4] Simonyan K and Zisserman A, “Very deep convolutional networks for large-scale image recognition,” 2014, arXiv:1409.1556.

[5] Han B, Sengupta A, and Roy K, “On the energy benefits of spiking deep neural networks: A case study,” in Proc. Int. Joint Conf. Neural Netw. (IJCNN), Jul. 2016, pp. 971–976.

[6] Hunsberger E and Eliasmith C, “Spiking deep networks with LIF neurons,” 2015, arXiv:1510.08829.

[7] Burkitt AN, “A review of the integrate-and-fire neuron model: I. Homogeneous synaptic input,” Biol. Cybern, vol. 95, no. 1, pp. 1–19, Jul. 2006. 16622699 10.1007/s00422-006-0068-6

[8] Rueckauer B, Lungu I-A, Hu Y, Pfeiffer M, and Liu S-C, “Conversion of continuous-valued deep networks to efficient event-driven networks for image classification,” Frontiers Neurosci, vol. 11, p. 682, Dec. 2017. 10.3389/fnins.2017.00682 PMC5770641 29375284

[9] Diehl PU, Neil D, Binas J, Cook M, Liu S-C, and Pfeiffer M, “Fast-classifying, high-accuracy spiking deep networks through weight and threshold balancing,” in Proc. Int. Joint Conf. Neural Netw. (IJCNN), Jul. 2015, pp. 1–8.

[10] Bu T, Fang W, Ding J, Dai P, Yu Z, and Huang T, “Optimal ANN-SNN conversion for high-accuracy and ultra-low-latency spiking neural networks,” 2023, arXiv:2303.04347.

[11] Sengupta A, Ye Y, Wang R, Liu C, and Roy K, “Going deeper in spiking neural networks: VGG and residual architectures,” Frontiers Neurosci., vol. 13, p. 95, Mar. 2019. 10.3389/fnins.2019.00095 PMC6416793 30899212

[12] Horowitz M, “1.1 computing’s energy problem (and what we can do about it),” in Proc. IEEE Int. Solid-State Circuits Conf. Dig. Tech. Papers (ISSCC), Feb. 2014, pp. 10–14.

[13] Rathi N and Roy K, “Diet-snn: A low-latency spiking neural network with direct input encoding and leakage and threshold optimization,” IEEE Trans. Neural Netw. Learn. Syst, vol. 34, no. 6, pp. 3174–3182, Jun. 2023. 34596559 10.1109/TNNLS.2021.3111897

[14] Deng S, Li Y, Zhang S, and Gu S, “Temporal efficient training of spiking neural network via gradient re-weighting,” in Proc. Int. Conf. Learn. Represent, 2022, pp. 1–17. [Online]. Available: https://openreview.net/forum?id=_XNtisL32jv

[15] Yao M, Hu J, Zhou Z, Li Y, Tian Y, Xu B, and Li G, “Spike-driven transformer,” in Proc. Adv. Neural Inf. Process. Syst, vol. 36, Oh A, Naumann T, Globerson A, Saenko K, Hardt M, and Levine S, Eds., 2023, pp. 64043–64058. [Online]. Available: https://proceedings.neurips.cc/paper_files/paper/2023/file/ca0f5358dbadda74b3049711887e9ead-Paper-Conference.pdf

[16] Chowdhury SS, Rathi N, and Roy K, “Towards ultra low latency spiking neural networks for vision and sequential tasks using temporal pruning,” in Proc. 17th Eur. Conf. Comput. Vis. (ECCV), Tel Aviv-Yafo, Israel. Berlin, Germany: Springer, 2022, pp. 709–726.

[17] Rawat W and Wang Z, “Deep convolutional neural networks for image classification: A comprehensive review,” Neural Comput., vol. 29, no. 9, pp. 2352–2449, Sep. 2017. 28599112 10.1162/NECO_a_00990

[18] Lenero-Bardallo JA, Serrano-Gotarredona T, and Linares-Barranco B, “A 3.6 *μ*s latency asynchronous frame-free event-driven dynamic-vision-sensor,” IEEE J. Solid-State Circuits, vol. 46, no. 6, pp. 1443–1455, Jun. 2011.

[19] Li Y, Geller T, Kim Y, and Panda P, “Seenn: Towards temporal spiking early exit neural networks,” in Proc. Adv. Neural Inf. Process. Syst, vol. 36, 2024, pp. 1–23.

[20] Xiao R, Yu Q, Yan R, and Tang H, “Fast and accurate classification with a multi-spike learning algorithm for spiking neurons,” in Proc. 28th Int. Joint Conf. Artif. Intell., Aug. 2019, pp. 1445–1451.

[21] Miao Y, Tang H, and Pan G, “A supervised multi-spike learning algorithm for spiking neural networks,” in Proc. Int. Joint Conf. Neural Netw. (IJCNN), Jul. 2018, pp. 1–7.

[22] Wang X and Zhang Y, “MT-SNN: Enhance spiking neural network with multiple thresholds,” 2023, arXiv:2303.11127.

[23] Feng L, Liu Q, Tang H, Ma D, and Pan G, “Multi-level firing with spiking DS-ResNet: Enabling better and deeper directly-trained spiking neural networks,” 2022, arXiv:2210.06386.

[24] Li H, Liu H, Ji X, Li G, and Shi L, “CIFAR10-DVS: An event-stream dataset for object classification,” Frontiers Neurosci., vol. 11, p. 309, May 2017. 10.3389/fnins.2017.00309 PMC5447775 28611582

[25] Orchard G, Jayawant A, Cohen GK, and Thakor N, “Converting static image datasets to spiking neuromorphic datasets using saccades,” Frontiers Neurosci, vol. 9, p. 437, Nov. 2015. 10.3389/fnins.2015.00437 PMC4644806 26635513

[26] Deng S and Gu S, “Optimal conversion of conventional artificial neural networks to spiking neural networks,” 2021, arXiv:2103.00476.

[27] Li Y, Deng S, Dong X, Gong R, and Gu S, “A free lunch from ANN: Towards efficient, accurate spiking neural networks calibration,” in Proc. Int. Conf. Mach. Learn., Jan. 2021, pp. 6316–6325.

[28] Neftci EO, Mostafa H, and Zenke F, “Surrogate gradient learning in spiking neural networks: Bringing the power of gradient-based optimization to spiking neural networks,” IEEE Signal Process. Mag, vol. 36, no. 6, pp. 51–63, Nov. 2019.

[29] Bellec G, Salaj D, Subramoney A, Legenstein R, and Maass W, “Long short-term memory and learning-to-learn in networks of spiking neurons,” in Proc. Adv. Neural Inf. Process. Syst, Jan. 2018, pp. 1–23.

[30] Deng L, Wu Y, Hu X, Liang L, Ding Y, Li G, Zhao G, Li P, and Xie Y, “Rethinking the performance comparison between SNNS and ANNS,” Neural Netw., vol. 121, pp. 294–307, Jan. 2020. 31586857 10.1016/j.neunet.2019.09.005

[31] Tan W, Patel D, and Kozma R, “Strategy and benchmark for converting deep Q-Networks to event-driven spiking neural networks,” in Proc. AAAI Conf. Artif. Intell., vol. 35, May 2021, pp. 9816–9824.

[32] Wang P, He X, Li G, Zhao T, and Cheng J, “Sparsity-inducing binarized neural networks,” in Proc. AAAI Conf. Artif. Intell., vol. 34, Apr. 2020, pp. 12192–12199.

[33] Rastegari M, Ordóñez V, Redmon J, and Farhadi A, “XNOR-Net: Imagenet classification using binary convolutional neural networks,” in Proc. Eur. Conf. Comput. Vis. Cham, Switzerland: Springer, 2016, pp. 525–542.

[34] Zhou S, Wu Y, Ni Z, Zhou X, Wen H, and Zou Y, “DoReFa-Net: Training low bitwidth convolutional neural networks with low bitwidth gradients,” 2016, arXiv:1606.06160.

[35] Yin P, Lyu J, Zhang S, Osher S, Qi Y, and Xin J, “Understanding straight-through estimator in training activation quantized neural nets,” 2019, arXiv:1903.05662.

[36] Wang Z, Fang Y, Cao J, and Xu R, “Adaptive calibration: A unified conversion framework of spiking neural networks,” 2023, arXiv:2311.14265.

[37] Li Y and Zeng Y, “Efficient and accurate conversion of spiking neural network with burst spikes,” 2022, arXiv:2204.13271.

[38] Datta G, Liu Z, and Beerel PA, “Can we get the best of both binary neural networks and spiking neural networks for efficient computer vision?” in Proc. The 12th Int. Conf. Learn. Represent., 2024, pp. 1–43.

[39] Miyashita D, Lee EH, and Murmann B, “Convolutional neural networks using logarithmic data representation,” 2016, arXiv:1603.01025.

[40] Lee EH, Miyashita D, Chai E, Murmann B, and Wong SS, “LogNet: Energy-efficient neural networks using logarithmic computation,” in Proc. IEEE Int. Conf. Acoust., Speech Signal Process. (ICASSP), Mar. 2017, pp. 5900–5904.

[41] Han S, Mao H, and Dally WJ, “Deep compression: Compressing deep neural networks with pruning, trained quantization and Huffman coding,” 2015, arXiv:1510.00149.

[42] Ioffe S and Szegedy C, “Batch normalization: Accelerating deep network training by reducing internal covariate shift,” in Proc. Int. Conf. Mach. Learn., Jan. 2015, pp. 448–456.

[43] Samadzadeh A, Far FST, Javadi A, Nickabadi A, and Chehreghani MH, “Convolutional spiking neural networks for spatio-temporal feature extraction,” Neural Process. Lett, vol. 55, no. 6, pp. 6979–6995, Dec. 2023.

[44] Li Y, Kim Y, Park H, Geller T, and Panda P, “Neuromorphic data augmentation for training spiking neural networks,” in Proc. Eur. Conf. Comput. Vis., Jan. 2022, pp. 631–649.

[45] Zheng H, Wu Y, Deng L, Hu Y, and Li G, “Going deeper with directly-trained larger spiking neural networks,” in Proc. AAAI Conf. Artif. Intell., vol. 35, May 2021, pp. 11062–11070.

[46] Kugele A, Pfeil T, Pfeiffer M, and Chicca E, “Efficient processing of spatio-temporal data streams with spiking neural networks,” Frontiers Neurosci, vol. 14, p. 439, May 2020. 10.3389/fnins.2020.00439 PMC7214871 32431592

[47] Wu Z, Zhang H, Lin Y, Li G, Wang M, and Tang Y, “LIAF-Net: Leaky integrate and analog fire network for lightweight and efficient spatiotemporal information processing,” IEEE Trans. Neural Netw. Learn. Syst, vol. 33, no. 11, pp. 6249–6262, Nov. 2022. 33979292 10.1109/TNNLS.2021.3073016

[48] Li Y, Guo Y, Zhang S, Deng S, Hai Y, and Gu S, “Differentiable spike: Rethinking gradient-descent for training spiking neural networks,” in Proc. Adv. Neural Inf. Process. Syst, vol. 34, Dec. 2021, pp. 23426–23439.

[49] Guo Y, Tong X, Chen Y, Zhang L, Liu X, Ma Z, and Huang X, “RecDis-SNN: Rectifying membrane potential distribution for directly training spiking neural networks,” in Proc. IEEE/CVF Conf. Comput. Vis. Pattern Recognit. (CVPR), Jun. 2022, pp. 326–335.

[50] Fang W, Yu Z, Chen Y, Masquelier T, Huang T, and Tian Y, “Incorporating learnable membrane time constant to enhance learning of spiking neural networks,” in Proc. IEEE/CVF Int. Conf. Comput. Vis. (ICCV), Oct. 2021, pp. 2641–2651.

[51] Wang K, Zhang J, Ren Y, Yao M, Shang D, Xu B, and Li G, “SpikeVoice: High-quality text-to-speech via efficient spiking neural network,” in Proc. 62nd Annu. Meeting Assoc. Comput. Linguistics, 2024, pp. 7927–7940.

[52] Xing X, Gao B, Zhang Z, Clifton DA, Xiao S, Du L, Li G, and Zhang J, “SpikeLLM: Scaling up spiking neural network to large language models via saliency-based spiking,” 2024, arXiv:2407.04752.

[53] Ding J, Bu T, Yu Z, Huang T, and Liu J, “SNN-RAT: Robustness-enhanced spiking neural network through regularized adversarial training,” in Proc. Adv. Neural Inf. Process. Syst, vol. 35, 2022, pp. 24780–24793.

[54] Ding J, Pan Z, Liu Y, Yu Z, and Huang T, “Robust stable spiking neural networks,” in Proc. 41st Int. Conf. Mach. Learn., vol. 235, Salakhutdinov R, Kolter Z, Heller K, Weller A, Oliver N, Scarlett J, and Berkenkamp F, Eds., Jul. 2024, pp. 11016–11029. [Online]. Available: https://proceedings.mlr.press/v235/ding24e.htm

[55] Lee J-J and Li P, “Reconfigurable dataflow optimization for spatiotemporal spiking neural computation on systolic array accelerators,” in Proc. IEEE 38th Int. Conf. Comput. Design (ICCD), Oct. 2020, pp. 57–64.

[56] Lee J-J, Zhang W, and Li P, “Parallel time batching: Systolic-array acceleration of sparse spiking neural computation,” in Proc. IEEE Int. Symp. High-Perform. Comput. Archit. (HPCA), Apr. 2022, pp. 317–330.

[57] Narayanan S, Taht K, Balasubramonian R, Giacomin E, and Gaillardon P-E, “SpinalFlow: An architecture and dataflow tailored for spiking neural networks,” in Proc. ACM/IEEE 47th Annu. Int. Symp. Comput. Archit. (ISCA), May 2020, pp. 349–362.

[58] Li J, Shen G, Zhao D, Zhang Q, and Zeng Y, “FireFly v2: Advancing hardware support for high-performance spiking neural network with a spatiotemporal FPGA accelerator,” IEEE Trans. Comput.-Aided Design Integr. Circuits Syst, vol. 43, no. 9, pp. 2647–2660, Sep. 2024.

[59] Intel Corporation. (2024). Intel 64 and IA-32 Architectures Software Developer’s Manual, Vol. 2A, Section 3-437. [Online]. Available: https://cdrdv2.intel.com/v1/dl/getContent/671200

[60] Krizhevsky A and Hinton G, “Learning multiple layers of features from tiny images,” Univ. Toronto, Tech. Rep, 2009. [Online]. Available: https://www.cs.utoronto.ca/kriz/learning-features-2009-TR.pdf

[61] DeVries T and Taylor GW, “Improved regularization of convolutional neural networks with cutout,” 2017, arXiv:1708.04552.

[62] Cubuk ED, Zoph B, Mané D, Vasudevan V, and Le QV, “AutoAugment: Learning augmentation strategies from data,” in Proc. IEEE/CVF Conf. Comput. Vis. Pattern Recognit. (CVPR), Jun. 2019, pp. 113–123.

[63] He K, Zhang X, Ren S, and Sun J, “Deep residual learning for image recognition,” in Proc. IEEE Conf. Comput. Vis. Pattern Recognit. (CVPR), Jun. 2016, pp. 770–778.

[64] He K, Zhang X, Ren S, and Sun J, “Delving deep into rectifiers: Surpassing human-level performance on ImageNet classification,” in Proc. IEEE Int. Conf. Comput. Vis. (ICCV), Dec. 2015, pp. 1026–1034.

